# Self-Assembling Amphiphilic ABA Triblock Copolymers of Hyperbranched Polyglycerol with Poly(tetrahydrofuran) and Their Nanomicelles as Highly Efficient Solubilization and Delivery Systems of Curcumin

**DOI:** 10.3390/ijms26125866

**Published:** 2025-06-19

**Authors:** Dóra Fecske, György Kasza, Gergő Gyulai, Kata Horváti, Márk Szabó, András Wacha, Zoltán Varga, Györgyi Szarka, Yi Thomann, Ralf Thomann, Rolf Mülhaupt, Éva Kiss, Attila Domján, Szilvia Bősze, Laura Bereczki, Béla Iván

**Affiliations:** 1 Polymer Chemistry and Physics Research Group, Institute of Materials and Environmental Chemistry, HUN-REN Research Centre for Natural Sciences, Magyar tudósok körútja 2, H-1117 Budapest, Hungary; 2 Hevesy György Doctoral School of Chemistry, ELTE Eötvös Loránd University, Pázmány Péter sétány 1/A, H-1117 Budapest, Hungary; 3 Laboratory of Interfaces and Nanostructures, Institute of Chemistry, Eötvös Loránd University, P.O. Box 32, H-1518 Budapest, Hungary; 4 MTA–HUN-REN "Momentum" Peptide-Based Vaccines Research Group, Institute of Materials and Environmental Chemistry, HUN-REN Research Centre for Natural Sciences, Magyar tudósok körútja 2, H-1117 Budapest, Hungary; 5 NMR Research Laboratory, Centre for Structural Science, HUN-REN Research Centre for Natural Sciences, Magyar tudósok körútja 2, H-1117 Budapest, Hungary; 6 Biological Nanochemistry Research Group, Institute of Materials and Environmental Chemistry, HUN-REN Research Centre for Natural Sciences, Magyar tudósok körútja 2, H-1117 Budapest, Hungary; 7 Freiburg Materials Research Center, University of Freiburg, Stefan-Meier-Str. 21, D-79104 Freiburg, Germany; 8 Freiburg Center for Interactive Materials and Bioinspired Technologies (FIT), University of Freiburg, Georges-Köhler-Allee 105, D-79110 Freiburg, Germany; 9 Institute for Macromolecular Chemistry, University of Freiburg, Stefan-Meier-Str. 31, D-79104 Freiburg, Germany; 10 HUN-REN–ELTE Research Group of Peptide Chemistry, Hungarian Research Network, Pázmány Péter sétány 1/A, H-1117 Budapest, Hungary; 11 Department of Genetics, Cell- and Immunobiology, Faculty of Medicine, Semmelweis University, Nagyvárad Tér 4, H-1089 Budapest, Hungary; 12 Chemical Crystallography Research Laboratory, Centre of Structural Science, HUN-REN Research Centre for Natural Sciences, Magyar tudósok körútja 2, H-1117 Budapest, Hungary

**Keywords:** hyperbranched polyglycerol (HbPG), poly(tetrahydrofuran) (PTHF), amphiphilic ABA triblock copolymer, self-assembly, nanomicelle, drug solubilization, drug delivery, nanomicellar carrier, curcumin, cell internalization

## Abstract

Delivering of hydrophobic drugs by polymeric nanoparticles is an intensively investigated research and development field worldwide due to the insufficient solubility of many existing and potential new drugs in aqueous media. Among polymeric nanoparticles, micelles of biocompatible amphiphilic block copolymers are among the most promising candidates for solubilization, encapsulation, and delivery of hydrophobic drugs to improve the water solubility and thus the bioavailability of such drugs. In this study, amphiphilic ABA triblock copolymers containing biocompatible hydrophilic hyperbranched (dendritic) polyglycerol (HbPG) outer and hydrophobic poly(tetrahydrofuran) (PTHF) inner segments were synthesized using amine-telechelic PTHF as a macroinitiator for glycidol polymerization. These hyperbranched–linear–hyperbranched block copolymers form nanosized micelles with 15–20 nm diameter above the critical micelle concentration. Coagulation experiments proved high colloidal stability of the aqueous micellar solutions of these block copolymers against temperature changes. The applicability of block copolymers as drug delivery systems was investigated using curcumin, a highly hydrophobic, water-insoluble, natural anti-cancer agent. High and efficient drug solubilization up to more than 3 orders of magnitude to that of the water solubility of curcumin (>1500-fold) is achieved with the HbPG-PTHF-HbPG block copolymer nanomicelles, locating the drug in amorphous form in the inner PTHF core. Outstanding stability of and sustained curcumin release from the drug-loaded block copolymer micelles were observed. The in vitro bioactivity of the curcumin-loaded nanomicelles was investigated on U-87 glioblastoma cell line, and an optimal triblock copolymer composition was found, which showed highly effective cellular uptake and no toxicity. These findings indicate that the HbPG-PTHF-HbPG triblock copolymers are promising candidates for advanced drug solubilization and delivery systems.

## 1. Introduction

Undoubtedly, one of the most challenging tasks of drug research and development is formulation of drugs with low water solubility to achieve efficient bioefficacy, due to the fact that many currently applied drugs and most of the new drug candidates are not soluble or have insufficient solubility in aqueous media. Among various possibilities, such as drug nanocrystals and liposome drug carriers, amphiphilic block copolymers continue to attract significant attention as drug solubilization, encapsulation, and delivery vehicles. In aqueous media, these block copolymers form nanomicelles due to self-assembly above the critical micelle concentration (*cmc*) and can solubilize hydrophobic agents in the core of the formed micelles [1,2,3,4]. The application of these self-assembling block copolymer nanocarriers as drug delivery systems has several advantages over other therapeutics, such as their high stability, controllable size, and the possibility of surface functionalization with imaging and/or targeting moieties [5,6,7,8,9]. In the last two decades, based on the beneficial properties of hyperbranched (dendritic) polymers, amphiphilic macromolecules with hyperbranched segments have also gained significant interest for biomedical applications [10,11,12,13,14]. One of the most attractive hyperbranched polymers for such purposes is hyperbranched polyglycerol (HbPG). HbPG has a variety of favorable properties, such as biocompatibility, low cytotoxicity, high membrane affinity, outstanding water solubility, and low viscosity [15,16]. Another advantage of HbPG is the simple and modular synthesis of its well-defined mono- and multifunctional derivatives [17,18,19,20,21]. Due to the biocompatibility of HbPG, its biomedical applications including drug delivery, bioimaging, theranostics, cancer treatment, and photodynamic therapy have been intensively investigated in recent years [22,23,24,25,26]. Polyglycerol-containing amphiphilic (co)polymers have also gained significant attention, and have been explored as drug and/or dye carriers. Unimolecular micelles and core–shell carriers with an HbPG core were synthesized by introducing hydrophobic groups, such as aromatic moieties and alkyl chains, into the HbPG core [27,28,29,30,31], or by grafting various polymer chains onto the HbPG core using post-polymerization or coupling methods [32,33,34,35,36]. Amphiphilic HbPG containing self-assembled carriers, modified with a smaller molecule or alkyl chain as the hydrophobic segment, have also been reported [37,38,39,40,41,42,43,44]. Polyglycerol-based amphiphilic copolymers were also prepared by using various linear hydrophobic blocks, such as poly(propylene oxide), poly(lactic acid), poly(ε-caprolactone), poly(*N*,*N*-diethylacrylamide), etc., and based on their self-assembling properties, these were applied as surfactants and drug delivery nanoparticles for various hydrophobic drugs [45,46,47,48,49,50,51,52,53,54,55,56].

Among hydrophobic components for amphiphilic block copolymers, poly(tetrahydrofuran) (PTHF), also called poly(tetramethylene oxide) (PTMO), is one of the most advantageous choices. PTHF is a biocompatible polymer, widely used in medical applications [57,58,59]. PTHF is commonly utilized in polyurethanes [60,61,62], epoxy resins [63,64], and (co)networks [65,66]. Amphiphilic block copolymers containing PTHF with various hydrophilic blocks, such as poly(ethylene oxide), poly(2-methyl-2-oxazoline), poly(α-amino acid)s, and poly(L-lysine), have also been reported [67,68,69,70,71]. These materials exhibit self-assembly in aqueous media and have been successfully used to encapsulate and release hydrophobic drugs. There is only one report [72] in which the synthesis of linear-branched PTHF-*b*-polyglycerol block copolymers with AB, ABA, and four-armed star structures and their micellar aggregation are claimed. However, the reported *cmc* values are questionably low compared to the previously published and similar PTHF- or HbPG-based block copolymers [45,47,68,69,70]. It is also mentioned in this publication [72] that these materials may be suitable for delivery systems, but did not provide any experimental result. Still, according to the best of our knowledge, drug solubilization, encapsulation, and release properties of the PTHF-HbPG block copolymers have not been investigated and reported so far.

In this study, we report on the synthesis of HbPG-PTHF-HbPG ABA-type hyperbranched–linear–hyperbranched triblock copolymers by the ring-opening, multibranching polymerization of glycidol using amine-telechelic PTHF as macroinitiator, followed by investigating its self-assembling properties, i.e., nanomicelle formation, the solubilization capacity of a drug having very low water solubility, its release, cytotoxicity, and cell internalization behavior. The block copolymers were synthesized with different monomer/macroinitiator ratios to investigate the effect of the composition on their properties and applicability. The HbPG-PTHF-HbPG block copolymers were explored as drug delivery systems by carrying out solubilization (encapsulation) and in vitro drug release studies of curcumin. This highly hydrophobic, natural, anti-tumor agent, which also has potent antiproliferative and apoptosis-inducing effects on several human cancer cell lines [73,74], possesses very low water solubility, that is, its solubilization and encapsulation are critical for biomedical applications (see, e.g., Refs. [56,75,76,77,78,79,80,81,82,83,84,85,86,87,88,89,90] and References therein). Herein, the biological activity of the curcumin-loaded nanomicelles of the amphiphilic HbPG-PTHF-HbPG block copolymers on the U-87 glioblastoma cell line is also reported to demonstrate their potential biomedical application possibilities.

## 2. Results and Discussion

### 2.1. Synthesis and Nanomicelle Formation of HbPG-PTHF-HbPG Block Copolymers

**Scheme 1:**
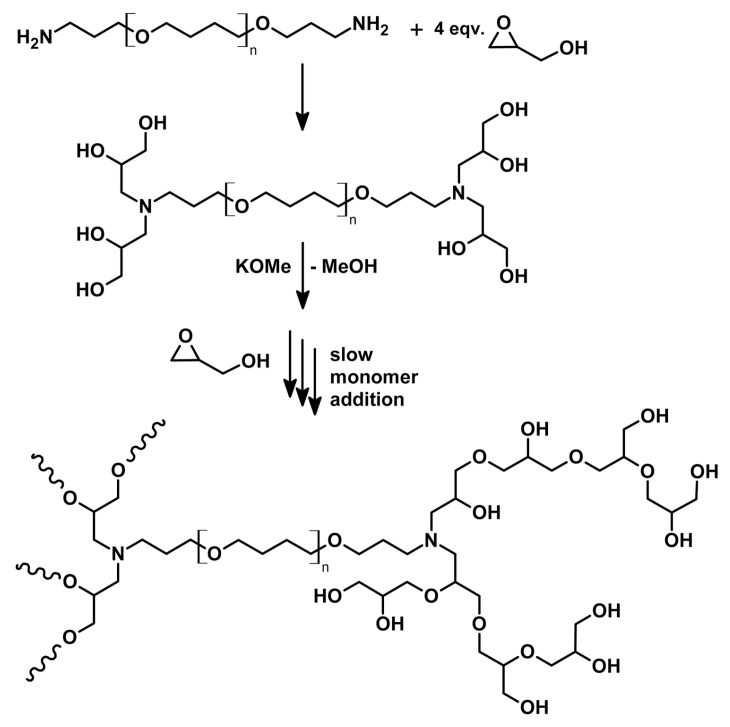
Synthesis of the HbPG-PTHF-HbPG amphiphilic ABA block copolymers.

The designed HbPG-PTHF-HbPG block copolymers were synthesized by the ring-opening, multibranching polymerization of glycidol by using an octahydroxyl-functional PTHF macroinitiator obtained by reacting four equivalents of glycidol with the amine-telechelic PTHF as displayed in Scheme 1. Three different monomer(M)/initiator(I) ratios, i.e., 15, 30, and 59 for samples ***P1***, ***P2***, and ***P3***, respectively, were applied to produce block copolymers with various hydrophilic/hydrophobic ratios and the same hydrophobic chain length (see also Experimental and Table 1). The resulting ABA block copolymers were characterized by several methods, such as ^1^H NMR spectroscopy, GPC, and DSC.

**Figure 1:**
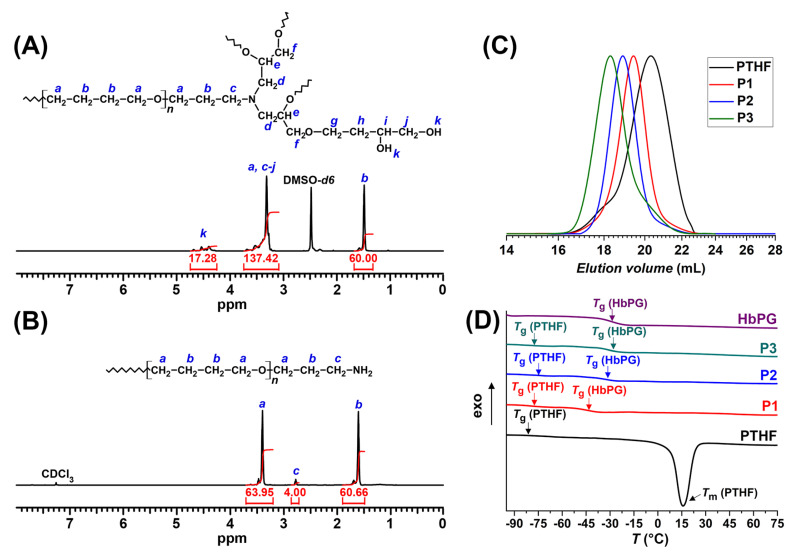
The ^1^H NMR spectra of the ***P1*** HbPG-PTHF-HbPG block copolymer ((**A**), in DMSO-*d*_6_) and the initial amine-telechelic PTHF ((**B**), in CDCl_3_). The GPC chromatograms of the HbPG-PTHF-HbPG amphiphilic block copolymers and the initial PHTF (**C**). DSC curves of the HbPG-PTHF-HbPG amphiphilic block copolymers, the initial PHTF, and the HbPG control sample synthesized by pentaerythritol initiator (**D**).

The ^1^H NMR spectra of the HbPG-PTHF-HbPG block copolymer ***P1*** and the amine-telechelic PTHF are shown in Figure 1A,B. In the ^1^H NMR spectrum of the starting amine-telechelic PTHF (Figure 1B), the signals for the aliphatic protons (-C*H*_2_-; 1.5–1.8 ppm), protons of the ether groups (-OC*H*_2_-; 3.2–3.7 ppm), and the methylene protons next to the amino end groups (-C*H*_2_-NH_2_; 2.7–2.85 ppm) were all observed. In the spectra of the block copolymers (see Figure 1A for ***P1*** and Figures S1 and S2 for the ^1^H NMR spectra of the ***P2*** and ***P3*** samples, respectively), the chemical shifts in the ether group protons of the PTHF and the HbPG main chain (-OC*H*_2_- and -OC*H*-) appeared to overlap in the region of 3.1–3.9 ppm. However, the signals of the aliphatic protons (-C*H*_2_-) of the PTHF and the hydroxyl protons of the HbPG are well separated in the 1.2–1.7 ppm and 4.2–4.7 ppm regions, respectively. Therefore, the *DP*_n_ of the HbPG component, calculated from the integrals of the ^1^H NMR signal of the hydroxyl groups of the HbPG blocks (*I*_OH_) relative to that of the PTHF’s aliphatic protons, are shown in Figure 1A, Figures S1, and S2 (considering the eight hydroxyl groups of the macroinitiator as well), and the number average molecular weights of the block copolymers (*M*_n,ABA_) can be determined as follows:

(1) $${DP}_{n}=I_{OH}-8$$

(2) $$M_{n,ABA}=\left({DP}_{n}+4\right)\cdot M_{glycidol}+M_{n,\;PTHF}$$

where *M*_glycidol_ is the molecular weight of the glycidol monomer and *M*_n,PTHF_ is the number average molecular weight of the initial PTHF determined from its ^1^H NMR spectrum. As presented in Table 1, the calculated number average molecular weights based on the *DP*_n_ values from the ^1^H NMR spectra agree well with the theoretical molecular weights (*M*_theor_).

To analyze the molecular weight distribution of the starting PTHF and the ABA block copolymers by GPC under the same conditions, the acetylation of the block copolymer samples was performed due to the fact that HbPG is insoluble in the THF eluent of the GPC. The acetylation was quantitative (>98%) in all cases, as demonstrated by ^1^H NMR measurements (see Figures S3–S5). The determined *M*_n_ values, based on the ^1^H NMR spectra of the acetyl derivatives of the block copolymers, are in a good agreement with the *M*_n_ of the unmodified polymers as shown in Table 1. The molecular weight distribution, the average molecular weights, and the dispersity indices (*Đ*) of the acetylated HbPG-PTHF-HbPG block copolymer derivatives and the starting amine-telechelic PTHF were measured by GPC. The GPC chromatograms are displayed in Figure 1C (for the molecular weight distribution curves see Figure S6), and the *M*_n_ and *Đ* results are presented in Table 1. As shown in Figure 1C, the GPC curves of the acetylated block copolymers are detected in lower elution volume regions than that of the initial PTHF, and the obtained *M*_n_ values follow an increasing trend with increasing monomer/initiator ratios. In addition, no significant peak appeared in the region of the initial PTHF in the GPC curves of the block copolymers, which confirms that the applied PTHF macroinitiator was completely incorporated into the polymer during the polymerization of glycidol. It has to be noted that the average molecular weights are relative values due to the difference in the hydrodynamic volumes of the homopolymer standards used for the calibration of GPC and that of the prepared HbPG-PTHF-HbPG block copolymers. Consequently, the *M*_n_ values determined from the ^1^H NMR results are applied in further calculations, instead of the indicative data obtained by using GPC measurements. The observed *Đ* values are relatively low (in the range of 1.3–1.6), but slightly increase with the increasing monomer/initiator ratio.

Figure 1D shows the DSC thermograms of the HbPG-PTHF-HbPG block copolymers as well as the starting PTHF and an HbPG homopolymer control sample (for the separated, full DSC curves, see Figures S7–S11). The obtained *T*_g_ values are shown in Table 1. The PTHF homopolymer has a glass transition temperature (*T*_g_) at −80 °C and a melting peak at 18 °C due to the semi-crystallinity of this homopolymer. The *T*_g_ of the HbPG homopolymer is −28 °C. The DSC curves of all the HbPG-PTHF-HbPG block copolymers exhibit two distinct glass transitions: one in the region of the *T*_g_ of the PTHF homopolymer and another one at around the *T*_g_ of the HbPG homopolymer. These findings clearly indicate phase separation of the hydrophilic HbPG and the hydrophobic PHTF segments in these ABA triblock copolymers. This result also confirms that the designed block copolymer structures are obtained. Furthermore, the *T*_g_ of the HbPG segment increases by increasing the molecular weight, but the *T*_g_ of the PTHF segment is not influenced significantly by the composition, that is, by the ratio of the hydrophobic/hydrophilic segments. However, the melting peak of the PTHF segment, which is visible in the DSC thermogram of the PTHF homopolymer in the range of 10–30 °C, does not appear in the DSC curves of the HbPG-PTHF-HbPG samples, i.e., crystallinity cannot be observed in the block copolymers (Figure 1D). This means that the crystallinity of the PTHF is significantly suppressed in the HbPG-PTHF-HbPG block copolymers, regardless of the size of the HbPG block and the ratio of the segments. Considering the ^1^H NMR, GPC, and DSC results, it can be concluded that the targeted HbPG-PTHF-HbPG triblock copolymers were successfully synthesized with the designed block structure and different hydrophobic/hydrophilic ratios.

**Figure 2:**
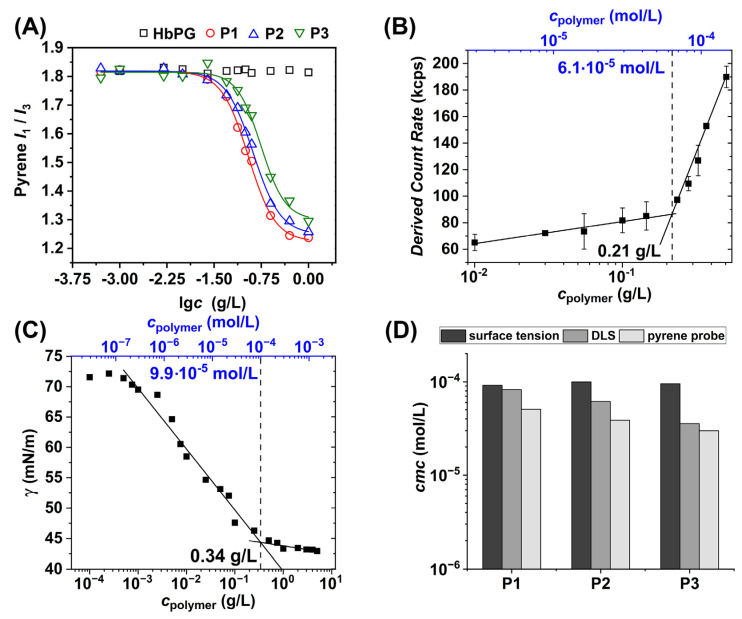
Pyrene *I*_1_/*I*_3_ values as a function of polymer concentration (**A**). Derived count rate determined by DLS (**B**) and surface tension (**C**) of the aqueous solutions of HbPG-PTHF-HbPG amphiphilic block copolymer (***P2***) as a function of the polymer concentration (the *cmc* value is marked with dashed line). The *cmc* values of the HbPG-PTHF-HbPG block copolymers obtained by the three different methods (**D**).

A block copolymer with different affinity segments exhibits amphiphilic characteristics and can self-assemble into micelle-like aggregates in aqueous media above the critical micelle concentration. The critical micelle concentrations (*cmc*) of the HbPG-PTHF-HbPG ABA triblock copolymers were determined by the well-established pyrene probe method [91]. No significant changes in the *I*_1_/*I*_3_ values were observed for the HbPG homopolymer control over the studied concentration range (Figure 2A). However, in the case of the block copolymers, a sharp decrease in the *I*_1_/*I*_3_ values was observed when the concentration was increased (Figure 2A). To resolve a specific concentration value of *cmc*, the measured points were fitted with a decreasing Boltzmann type sigmoid, which is given by

(3) $$y=\frac{A_{1}-A_{2}}{1+e^{(x-x_{0})/∆x}}+A_{2}$$

where the variable *y* corresponds to the *I*_1_/*I*_3_ values, while *x* is the total block copolymer concentration. According to Aguiar et al., the *cmc* should be given as the center of the sigmoid (*x*_0_) if the ratio *x*_0_/Δ*x* is smaller than 10 and as (*x*_0_ + 2Δ*x*) otherwise [91]. Since the *x*_0_/Δ*x* < 10 condition was valid in all cases, *x*_0_ was selected as *cmc*. As shown in Table 2, the *cmc* values of the HbPG-PTHF-HbPG block copolymers are in the range of 0.1–0.2 g/L, which are in good agreement with the reported *cmc* values for similar HbPG-based block copolymers, such as poly(propylene oxide)-*b*-HbPG [45,47]. The *cmc* values previously reported for PTHF-containing block copolymers vary in a wide range, i.e., ~0.005–0.005 mg/mL for PTHF-PEG [67], poly(2-methyl-2-oxazoline), and PTHF-based ABA block copolymer [69], ~0.05–0.1 mg/mL for ABA block copolymers of poly(L-lysine) and PTHF [68], and ~0.9–8 mg/mL for block copolymers of different hydrophilic poly(oxazoline)s and PTHF [70]. The differences in the *cmc* values for different PTHF-containing block copolymers are due to the fact that the *cmc* is mainly determined by the length of the hydrophobic block [2,10]. Therefore, comparison of these *cmc* values is uninformative.

There is only one study, which reports on the attempt for the synthesis of PTHF-HbPG block copolymers and the determination of their *cmc* values [72]. However, hydroxyl-ended PTHFs were used as macroinitiators, leading to less than quantitative initiation of glycidol, and thus to the presence of unreacted PTHF as described in this publication. Therefore, taking into account this fact and the lack of the presentation of sufficient experimental evidence for the formation of the claimed copolymer structures, it is highly questionable whether the reported *cmc* values, which are in the range of 0.8−3.0·10^−3^ g/L, really belong to the depicted block copolymer formulas as presented in this report [72].

To clarify this contradiction, the *cmc* values were determined by us as well as with two other methods. The derived count rate, determined by DLS measurements of the aqueous solutions of ***P2***, is plotted as a function of the polymer concentration in Figure 2B (for the ***P1*** and ***P3*** copolymers, see Figure S12). As shown in Figure 2B, the derived count rate values are low (~60–80 kcps) at low polymer concentrations and increase steeply at higher polymer concentrations due to micelle formation. The *cmc* values of the copolymers are defined as the intersection of the fitted lines, and these are presented in Table 2. The surface activity of the prepared HbPG-PTHF-HbPG block copolymers was characterized by measuring the surface tension of their aqueous solution by the Du Noüy ring method as a function of concentration in the range of 10^−4^−5 g/L. As displayed in Figure 2C, the surface tension values, plotted for a representative sample (***P2***), decrease even at low polymer concentrations, indicating a strong tendency for surface adsorption of the macromolecules due to their amphiphilic nature. The shape of the curve is typical of a surfactant, i.e., the surface tension decreases as the polymer concentration increases, and then reaches a low value, from which it does not decrease further. As shown in Figure 2C, the *cmc* can be obtained by determining the intersection of the lines fitted to the data in the decreasing and lower plateau regions of the surface tension versus concentration plots (for the surface tension versus polymer concentration data of samples ***P1*** and ***P3***, see Figure S13). The determined *cmc* values in g/L and mol/L are presented in Table 2. The *cmc* is lower than 1 g/L for all the HbPG-PTHF-HbPG block copolymers and decreases approximately linearly with increasing PTHF content (***P1***: 0.20 g/L; ***P2***: 0.34 g/L; and ***P3***: 0.56 g/L); however, the *cmc* values in mol/L are almost the same. This concentration range corresponds well to that determined by the pyrene probe method and DLS measurements. The observed trend is also similar, although the *cmc* values determined from the surface tension measurements are slightly higher than those determined by the pyrene probe method and DLS.

**Figure 3:**
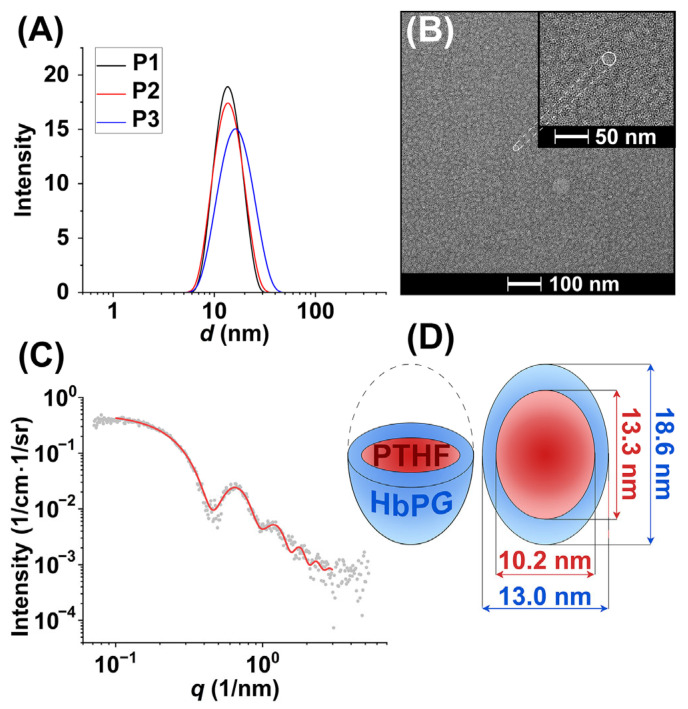
The DLS curves of the HbPG-PTHF-HbPG block copolymer micelles (**A**). TEM images of ***P2*** block copolymer-based micelles (**B**). The scattering curve of the ***P1*** block copolymer-based micelles (**C**) and a schematic illustration of the formed micelles according to SAXS measurement (**D**).

The size and morphology of the formed micellar-type aggregates were determined by DLS, TEM, and SAXS measurements (see Figure 3 and Table 2). According to the DLS measurements, the hydrodynamic diameter of the HbPG-PTHF-HbPG block copolymers is in the range of 13–15 nm with a narrow size distribution (Figure 3A). The TEM measurement of the ***P2*** sample shows micelles with an average size of 15.1 ± 2.5 nm (see Figure 3B, Figures S14, and S15), which supports the DLS results. The SAXS curve of the ***P1*** sample in Figure 3C can be fitted quite well by a core–shell ellipsoid model, according to which a schematic illustration is presented in Figure 3D. The micelle size of 18.6 nm from the SAXS curves agrees well with the results obtained by DLS and TEM measurements. Therefore, it can be concluded that the HbPG-PTHF-HbPG block copolymers self-assemble into nanosized micelles with a hydrodynamic diameter between 13 and 20 nm above their *cmc*. These nanosized micelles show excellent colloidal stability over a wide temperature range, i.e., thermally induced macroscopic aggregation does not occur even at high temperatures (Figures S16–S18), demonstrating that these materials are promising candidates for biomedical applications.

### 2.2. HbPG-PTHF-HbPG Block Copolymer Nanomicelles as Drug Solubilization and Delivery Systems

The applicability of the prepared amphiphilic HbPG-PTHF-HbPG block copolymers as drug delivery systems was investigated via solubilization (encapsulation) and in vitro drug release studies of curcumin. This compound is a highly hydrophobic bioactive molecule with potent cytotoxicity against a variety of cancer cells, as demonstrated by in vitro studies [86,87]. It is important to note that the low aqueous solubility of curcumin leads to poor bioavailability, which is the major limiting factor for its use as a therapeutic agent. To overcome this problem, curcumin was dissolved in several cases in organic solvents, such as DMSO, ethanol, or methanol, which cannot be used in animal or in vivo clinical studies due to their toxicity [86]. Others applied polymeric micelles, which afforded to use aqueous systems for curcumin delivery [87]. In our case, curcumin was solubilized by the HbPG-PTHF-HbPG block copolymer nanomicelles via partitioning between the micelles in aqueous solution and curcumin, added as solution in acetone and followed by the evaporation of acetone, led to nanomicelles with encapsulated curcumin in an aqueous medium.

**Figure 4:**
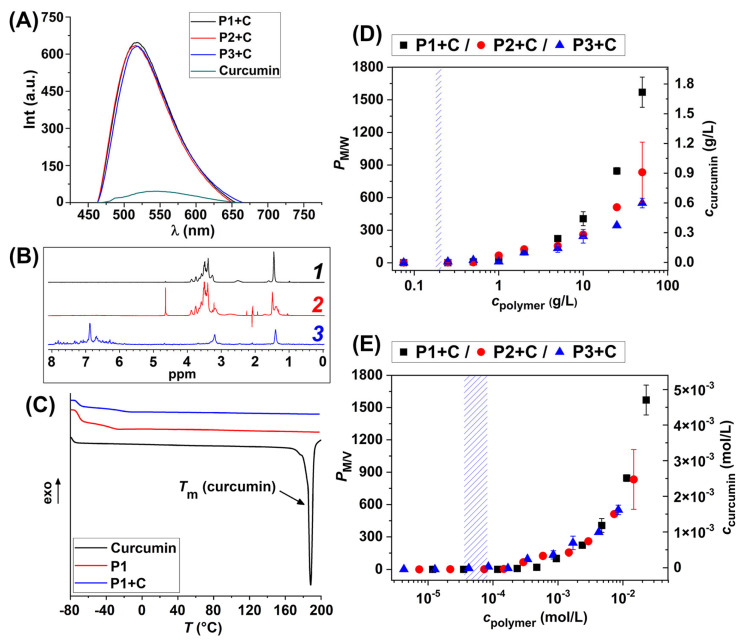
Fluorescence spectra of the curcumin-loaded HbPG-PTHF-HbPG nanomicelles and curcumin (**A**). ^1^H NMR spectra of the curcumin-free block copolymer ***P1*** ((**B**), 1), ^1^H NMR ((**B**), 2), and ^1^H dpfgse NOESY (irradiation at 7.14 ppm) ((**B**), 3) spectra of curcumin-loaded HbPG-PTHF-HbPG micelles in D_2_O at 25 °C. The DSC thermograms of curcumin, ***P1***, and curcumin-loaded ***P1*** micelles (**C**). Micelle/water partition coefficient (*P*_M/W_) and curcumin concentration as a function of mass (**D**) and molar (**E**) concentration of the HbPG-PTHF-HbPG amphiphilic block copolymers (the marked areas denote the region of the *cmc*).

The fluorescence emission spectra of the free curcumin and the curcumin-loaded micelles are presented in Figure 4A. In the fluorescence spectra of curcumin, there is a weak broad peak at 550 nm, but the curcumin-loaded micelles show a well-defined, high intensity blue-shifted peak at 520 nm, suggesting that the curcumin is located in the hydrophobic core, as previously reported for curcumin encapsulation by polymeric micelles [88,89]. To confirm this, the polymer–drug interactions were studied using solution state NMR spectroscopy. There are three possible sites for curcumin in the nanomicelle: (i) in the middle of the micelles solvated by the hydrophobic PTHF part of the block copolymers; (ii) both in the PTHF core and HbPG shell; (iii) only in the outer sphere of the micelles solvated by the hydrophilic HbPG. Based on the comparison of the ^1^H NMR spectra of the curcumin-free and -loaded micelles (see Figure 4B, spectra 1 and 2), two broad signals (1.41 and 3.20 ppm) appear in the spectra of the latter. The PTHF chains are stress-free in the unloaded micelles, that is, all the chains are in the lowest energy state. When drug molecules are incorporated into the micelles, the chains have to populate the gauche conformations as well. This effect results in the appearance of slightly shifted and broader signals. In addition, broad signals of the curcumin indicate fast *T*_2_ relaxation (typical for large molecules), which can be attributed to the fact that it is incorporated in the micelles. The curcumin-loaded polymeric micelles were studied by one-dimensional NOESY (dpfgse) measurements, and the spectrum was obtained by selectively irradiating the signals of curcumin at 7.14 ppm (Figure 4B, spectrum 3). Intermolecular NOE signals of PTHF appeared at 1.60 and 3.40 ppm, similar to the two-dimensional spectrum (see Figure S19). This suggests spatial proximity of less than 5 Å indicates that curcumin is incorporated in the hydrophobic PTHF part of the micelle. Moreover, only very weak and broad signals appear in the 3.8–3.9 ppm region, indicating low interaction between the drug and the HbPG shell of the micelles. The NOE signals have the same phase as the irradiated one because the autocorrelation time of the complex falls within the range of negative NOE effect at this field strength. Based on the results of the fluorometric and NOESY measurements, we can conclude that most of the solubilized curcumin is incorporated in the PTHF core of the nanomicelles, and the effect of the HbPG shell is very slight. Additionally, the analysis of the thermal properties of the curcumin in free and encapsulated form proves that the solubilized curcumin is in an amorphous state in the micelles, as the curcumin melting peak at 188 °C cannot be observed in the DSC thermogram of the curcumin-loaded ***P1*** block copolymer (Figure 4C), which is in accordance with results reported for curcumin–zein particles [90].

The concentration of the curcumin in the HbPG-PTHF-HbPG aqueous solutions with a wide polymer concentration range was measured by UV-Vis spectroscopy, and the obtained results are presented in Figure 4D,E. Based on the determined curcumin concentrations, the micelle/water partition coefficient (*P*_M/W_) and drug loading content (*DLC*) of curcumin were determined. The *P*_M/W_ values and the solubilized curcumin concentration versus the applied block copolymer concentration in g/L and in mol/L are displayed in Figure 4D and Figure 4E, respectively (the *DLC* as a function of the polymer concentration is presented in Figure S20). As shown in these figures, solubilized drug concentrations and the *P*_M/W_ values increase with increasing polymer concentration for all the block copolymers, but only above the *cmc* (*c*_polymer_ > 0.2 g/L). Above the *cmc* in the range of 1–5 g/L polymer concentration, the amount of solubilized curcumin is almost the same for all three block copolymers. However, at higher mass concentrations there are significant differences between the copolymers; namely, the drug uptake decreases with increasing molecular weight, i.e., with the increasing size of the hydrophilic HbPG segment. The *P*_M/W_ value decreases as the molecular weight of the block copolymer increases. This tendency is also observed for the *DLC* values in g/g. These findings are well presented by the data in Table 3 for the curcumin solubilization (encapsulation) characteristics of the HbPG-PTHF-HbPG block copolymers at 50 g/L polymer concentration. It should also be emphasized that at a polymer concentration of 50 g/L, the poor water solubility of curcumin was increased by more than 1500-fold in the case of the ***P1*** polymer. Since the self-assembly behavior is mainly determined by the PTHF segment, it is worth examining the evolution of *P*_M/W_ as a function of the molar concentration of the copolymers as well (Figure 4E). As shown in this figure, the drug uptake is nearly the same at higher polymer concentrations for all the block copolymers, which is also supported by the *DLC* expressed in mol/mol (Figure S20B). For comparison, in the case of similar polymeric micellar drug delivery systems investigated recently, such as block copolymers of PEG with polycaprolactone (PCL) or poly(lactic acid) (PLA), *DLC* values in a relatively wide range of ~1–15% are reported for curcumin encapsulation [92,93,94,95,96,97,98]. Consequently, the *DLC* values obtained for the HbPG-PTHF-HbPG block copolymers are similar to those reported for other curcumin delivery systems based on polymer micelles. However, in these reports, the drug encapsulation is studied only with one polymer concentration, and its effect on the stability of the micelles and drug encapsulation properties are not presented as investigated and discussed herein.

**Figure 5:**
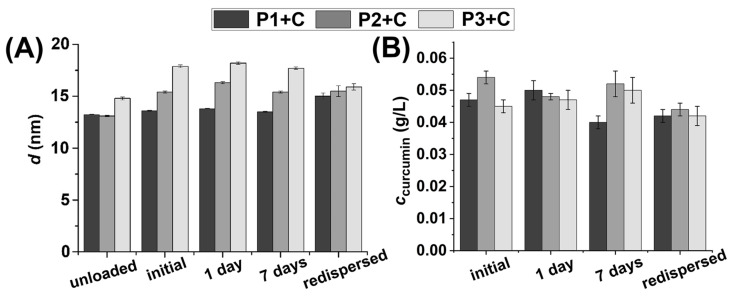
The size (**A**) and the drug content (**B**) of curcumin-loaded and unloaded micelles of the HbPG-PTHF-HbPG block copolymers after preparation, 1 and 7 days storage, and after lyophilization followed by redispersion.

The average hydrodynamic particle size and dispersity of the curcumin-loaded HbPG-PTHF-HbPG micelles were determined by DLS (for DLS curves and the *d* and *Đ* values, see Figures S21–S23 and Table S1 in the Supporting Information). It was found that the average hydrodynamic diameter slightly increased by curcumin-loading, i.e., 13.6 nm (*Đ* = 0.040), 15.4 nm (*Đ* = 0.089), and 17.9 nm (*Đ* = 0.138) by increasing the molecular weight and the hydrophilic HbPG content, respectively (Figure 5A). As observed, the hydrodynamic diameter due to drug loading is slightly increased by the increasing size of the HbPG segment. Still, it has no significant effect on the dispersity of the nanosized formulations. The colloidal stability of the curcumin-loaded HbPG-PTHF-HbPG nanomicelles was also investigated by DLS and UV-Vis spectroscopy (Figure 5A,B; for DLS curves and curcumin concentrations, see Figures S21–S23 and Table S1). As the presented data in these figures indicate, the size and the curcumin content of the drug-loaded micelle solutions were almost constant for over 7 days. This result confirms that the curcumin-loaded micelles have high stability in aqueous solution. From the pharmaceutical technology and drug formulation perspective, dry storage and redispersibility are critical parameters. Therefore, the size and drug content of lyophilized samples, stored at ambient conditions for 3 days and redispersed in water, were also examined (Figure 5A,B). As obtained, the size and the curcumin content did not change significantly after lyophilization and redispersion compared to the initial curcumin-loaded micelles. This finding proves significant drug storage stability of the curcumin/HbPG-PTHF-HbPG formulation with even relatively high solubilized (encapsulated) curcumin content.

**Figure 6:**
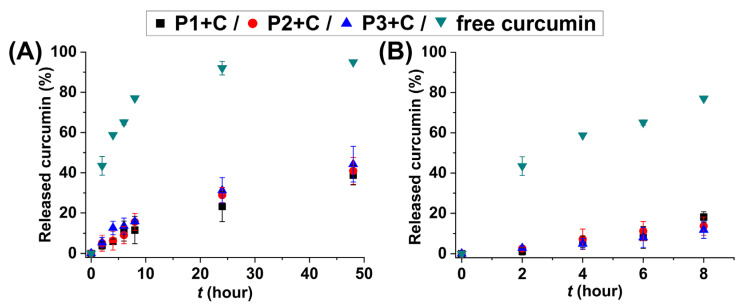
Curcumin release of the HbPG-PTHF-HbPG amphiphilic block copolymers and free curcumin: 1 g/L curcumin and 25 g/L polymer solutions (**A**) and 0.1 g/L curcumin and 1.7 mmol/L polymer solutions (**B**) were used for the preparation of the curcumin-loaded block copolymer nanomicelles.

The release of curcumin from the loaded polymer micelles was measured by the dialysis method. Figure 6A shows that a relatively rapid transfer of free curcumin, dissolved in DMSO/water (40/60 *V*/*V*%), occurs across the dialysis membrane, which proves that this method can be effectively used to study the curcumin release from the HbPG-PTHF-HbPG triblock copolymer nanomicelles. As can be seen in Figure 6A,B, the HbPG-PTHF-HbPG nanomicelles decelerate the release of curcumin. The drug release profile follows a similar pattern for the polymeric micelles: An initial fast release in the first 8 h followed by a more gradual release phase, and 20–30% of the encapsulated curcumin was released after 24 h for all three block copolymers. The mechanism of the drug release was evaluated on the basis of the known release kinetic models, and it was found that the long-term release data fitted well only with the widely used Korsmeyer–Peppas equation [99] (Figure S24). This resulted in *n* values in the range of 0.53–0.56, indicating a Fickian diffusion drug release mechanism of curcumin from the HbPG-PTHF-HbPG nanomicelles. In the first 8 h, there was no significant difference between the drug release rates of the block copolymers. Still, it appears, thereafter, that the amount of curcumin released from the micelles is slightly increased by the increasing molecular weight of the HbPG-PTHF-HbPG samples. This is thought to be due to differences in the initial amount of solubilized drug and the composition of the block polymers (molecular weight and hydrophobic/hydrophilic ratio). Therefore, to understand the effect of the composition of the HbPG-PTHF-HbPG block copolymers on the curcumin release, the drug release experiments were repeated for 8 h, which is highly relevant in the circulatory system, using the same concentration of the polymers (1.7 mmol/L) and drug (0.27 mmol/L; 0.1 g/L). The results in Figure 6B show that the curcumin release profiles follow an identical and nearly linear trend, although some difference between the released amount of curcumin from the different block copolymers can be observed at 8 h of incubation time. Namely, the released curcumin is 18% using block copolymer ***P1*** (50% hydrophobic PTHF content), while it is around 11% in the case of the ***P3*** block copolymer (20% hydrophobic PTHF content). The effect of HbPG segment size can be explained by the well-hydrated outer glycerol units of the HbPG, which form a dense shell layer and can block the release of the guest curcumin molecules. This result agrees with previous data published by Wu and coworkers [100], who found slower release of hydrophobic guest molecules when using high molecular weight HbPG, as well as improved release properties by selective modification of the hydroxyl end groups. Based on this result, it can be concluded that the hydrophobic PTHF segment primarily determines the drug uptake and release properties of HbPG-PTHF-HbPG block copolymers, but increasing the size of the hydrophilic HbPG segment slightly delays the release.

Particle size is critical for the biodistribution and cellular uptake properties of polymeric micelles [101], as their small size (10–200 nm) allows them to accumulate in tumor cells due to the enhanced permeation and retention (EPR) effect [102]. In addition, the micelles, which are not larger than 100 nm, have significant potential for delivering drugs across the blood–brain barrier [103]. Hence, the bio-applicability of the prepared HbPG-PTHF-HbPG triblock copolymers was tested on U-87 glioblastoma cell line, which is commonly used in brain cancer research.

**Figure 7:**
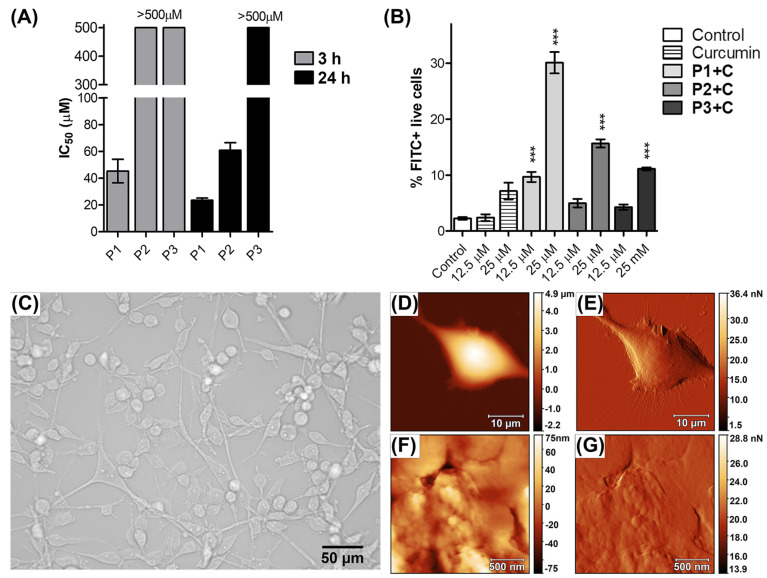
The cytotoxicity of the block copolymers on U-87 glioblastoma cells incubated with the polymers for 3 and 24 h. Bars represent mean *IC*_50_ values ± SEM, n = 4 (**A**). Internalization degree of curcumin and curcumin-loaded micelles of U-87 glioblastoma cells after 3 h treatment with curcumin and ***P1*** + ***C***, ***P2*** + ***C***, ***P3*** + ***C*** curcumin-loaded block copolymers. Percentage of FITC-positive live cells (**B**) is presented. Data are mean ± SEM for n = 3. *** *p* < 0.0001. During the flow cytometric measurement, relative viability of the compound-treated cells was over 78% compared to untreated control cells. Bright field microscopic image, captured by Olympus CKX objective 10X (**C**), AFM topography (**D**), and cantilever deflection (**E**) images of curcumin-treated and glutaraldehyde-fixed U-87 cells. Surface roughness was determined at the central region of the cells; high-resolution images of the same area are presented in panel (**F**) (topography) and panel (**G**) (deflection image). Similar images were obtained for ***P1*** + ***C***, ***P2*** + ***C***, and ***P3*** + ***C*** polymers.

To assess the bioavailability of the block copolymers, the micelles were tested for their cytotoxicity on U-87 glioblastoma cells before curcumin loading. A short- and long-term treatment was tested, that is, 3 and 24 h incubation periods were assayed. As shown in Figure 7A, the ***P3*** polymer is not cytotoxic even at the highest test concentration (500 µM), while ***P2*** causes cell death only after 24 h of treatment. ***P1*** polymer showed moderate cytotoxicity after 3 h and 24 h of incubation. The *IC*_50_ values of the ***P1*** polymer were 45.3 and 23.5 µM, respectively. Based on the *IC*_50_ values, it can be assumed that there is a minimum HbPG content required to avoid the cytotoxic effect of the unloaded block copolymers.

Cellular uptake of curcumin-loaded micelles on U-87 glioblastoma cells was measured by flow cytometry after 3 h of treatment with curcumin and curcumin-loaded micelles (***P1*** + ***C***, ***P2*** + ***C***, ***P3*** + ***C***). None of the compounds showed relevant cytotoxicity to U-87 cells in the concentration range and incubation time used. Nevertheless, the highest cellular uptake was measured for the same ***P1*** + ***C*** sample. At both concentrations, the internalization rates for all curcumin-loaded block copolymers were significantly higher than the internalization rate of free curcumin. As can be seen in Figure 7B, increasing the HbPG content of the block copolymers decreases the cellular uptake of U-87 cells. However, it is essential to note that the mean fluorescence signal intensity was measured after trypsinization. Trypsinization detaches adherent cells from the plate in which they were treated to transfer them into FACS tubes. During trypsinization, cell surface proteins and “non-specific signal” (caused by surface-bound fluorescent compounds) are digested and removed from the cell surface. Therefore, in the flow cytometric analysis the fluorescent signal resulted in the internalized, intracellular amount of the HbPG-PTHF-HbPG block copolymers.

Based on the microscopic and AFM images, no severe damage or morphological differences were observed after 3 h of treatment with curcumin and curcumin-loaded micelles at a concentration of 25 µM. High-resolution images of the glutaraldehyde-fixed cells were recorded using AFM. Representative images of cells at two levels of resolution are shown in Figure 7D–G, and the average surface roughness values (*R*_α_) determined are listed in Table 4**.**

A slight decrease in the average *R*_α_ values was observed for all compound-treated U-87 cells compared to the untreated control. However, except for the ***P3*** + ***C***-treated cells, these changes cannot be considered as statistically significant. Membrane roughness is a sensitive parameter that reflects the incorporation of a material into the lipid layer of neuronal cells and provides a reliable, label-free method to monitor the changes at the cell surface [104,105]. Because polymer ***P3*** has the highest molecular weight and contains the largest HbPG segments, the reduced membrane roughness can be attributed to an increase in the membrane pressure caused by the incorporation of the polymer.

## 3. Materials and Methods

### 3.1. Materials

Glycidol (from Sigma-Aldrich, Steinheim, Germany) was distilled under reduced pressure before use. Bis(3-aminopropyl)-terminated poly(tetrahydrofuran) (*M_n_* = 1100 g/mol) (from Sigma-Aldrich, Steinheim, Germany) was purified by precipitation from THF into hexane and dried until constant weight in vacuum at 40 °C. Acetic anhydride (99%), ethanol, acetone, diethyl ether, hexane, potassium methoxide solution (25 % in methanol), pyridine, (all from Molar Chemicals Ltd., Halásztelek, Hungary), pyrene, Tween 80, MTT (Thiazolyl Blue Tetrazolium Bromide) (all from Sigma-Aldrich, Steinheim, Germany), glutaraldehyde 25% (from Merck, Darmstadt, Germany), PBS (pH = 7.4) (from Lonza, Basel, Switzerland), and curcumin (from Alfa Aesar, Ward Hill, MA, USA) were used as received without purification. Tetrahydrofuran (THF, from Molar Chemicals Ltd., Halásztelek, Hungary) was refluxed over and distilled from potassium hydroxide before being used as an eluent in gel permeation chromatography. Double-distilled water, checked by its electrical conductivity (<5 mS) and surface tension (72.0 mN/m at 25 ± 0.5 °C), was used to prepare the block copolymer solutions. Dulbecco’s Modified Eagle Medium (DMEM) was supplemented with 10% (*v*/*v*) Fetal Bovine Serum (FBS), l-glutamine (2 mM), sodium pyruvate (1 mM), penicillin–streptomycin (250 units potassium penicillin and 250 μg streptomycin sulfate) (all from Lonza, Basel, Switzerland), and non-essential amino acids (NEAA, from Sigma-Aldrich, Steinheim, Germany). HPMI was prepared in-house from salts obtained from Sigma-Aldrich (Steinheim, Germany) (100 mM NaCl, 5.4 mM KCl, 0.4 mM MgCl_2_, 0.04 mM CaCl_2_, 10 mM Hepes, 20 mM glucose, 24 mM NaHCO_3_, and 5 mM Na_2_HPO_4_ at pH 7.4) [106]. Hyperbranched polyglycerol (*M*_n_ = 2460 g/mol; for NMR spectrum, see Figure S25) as a control sample for DSC and fluorometric studies was synthesized in our laboratory according to our previously published method using neopentyl alcohol as the initiator [38].

### 3.2. Synthesis of Hyperbranched Polyglycerol (HbPG) and Poly(tetrahydrofuran) (PTHF) Containing ABA Triblock Copolymers

The HbPG-PTHF-HbPG triblock copolymers were synthesized by bulk ring-opening, multibranching polymerization of glycidol using bis(3-aminopropyl)-telechelic poly(tetrahydrofuran) (NH_2_-PHTF-NH_2_) as an initiator and three different monomer (*M*)-to-macroinitiator (*I*) ratios. The synthesis of the HbPG-PTHF-HbPG block copolymer with a theoretical molecular weight (*M*_theor_) of 2200 g/mol (***P1***: *M*/*I* = 15) is described below. First, the bis(3-aminopropyl)-telechelic poly(tetrahydrofuran) (6.8349 g, 5.6 mmol) and four equivalents of glycidol (1.62 mL, 24.4 mmol) were placed into a 100 mL three-neck round-bottom flask equipped with a mechanical stirrer, connected to a vacuum line, with the third neck sealed by a septum. The reaction flask was flushed with nitrogen and the inert atmosphere was obtained by repeated pump–thaw cycles five times. The mixture was then heated to 55 °C and stirred for one hour. Subsequently, the appropriate amount of potassium methoxide (1.44 mL, 19.5 mmol) was added, and the mixture was stirred at the same temperature for one hour. Then, the methanol was removed by vacuum and the reaction flask was heated to 95 °C, and 4.28 mL of glycidol monomer (4.7708 g, 64.4 mmol) was added to the flask using a syringe pump (feed rate: 3 mL/h). After the monomer addition, the stirring was continued for another 2 h. The cooled crude product was dissolved in 40 mL ethanol and passed through a column packed with cation exchange resin (Amberlite IR120, hydrogen form). The solution was concentrated with a rotary evaporator and precipitated into a large excess of diethyl ether/hexane (1:1 *V/V*). The collected polymer was dried to constant weight in a vacuum oven at 55 °C. The other two block copolymers were prepared similarly but with different monomer/initiator ratios (***P2***: *M*/*I* = 30, *M*_theor_ = 3300 g/mol; ***P3***: *M*/*I* = 59, *M*_theor_ = 5500 g/mol). The obtained yields were 91, 90, and 85%, respectively.

### 3.3. Characterization Methods

#### 3.3.1. Gel Permeation Chromatography

Acetylated derivatives of the block copolymers were synthesized to investigate the molecular weight and molecular weight distribution by gel permeation chromatography (GPC) [17]. For the acetylation, 300 mg of HbPG-PTHF-HbPG block copolymer was measured into a 25 mL round-bottom flask and dissolved in 8 mL pyridine. Then, the solution was heated to 75 °C under an inert atmosphere, and 1 mL of acetic anhydride was added to the solution. This reaction mixture was stirred at 75 °C overnight. The solvent and the byproducts were then removed by a rotary evaporator. The crude product was dissolved in 10 mL DCM and washed three times with 5 mL water. Then, DCM was removed by a rotary evaporator and the polymers were dissolved in 3 mL of THF and precipitated into a large excess of hexane. The products were collected by centrifugation and then dried at 55 °C under vacuum to constant weight. The acetylated HbPG-PTHF-HbPG block copolymers and the initial NH_2_-PTHF-NH_2_ were analyzed by GPC equipped with a Waters 717+ Autosampler injector (Waters, Milford, MS, USA), a Waters 515 HPLC pump (Waters, Milford, MS, USA), a Waters column set (Waters, Milford, MS, USA; HR1, HR4 columns and a Waters guard column) thermostated at 35 °C by a JetStream column thermostat, and an Agilent 1260 Infinity RI detector (Agilent Technologies, Santa Clara, CA, USA). PSS WinGPC software, version 8.40 (Polymer Standard Service, Mainz, Germany) was used to evaluate the measurements. The mobile phase was THF with a 1 mL/min flow rate. The evaluation of the chromatograms was based on calibration with linear polystyrene standards.

#### 3.3.2. NMR Spectroscopy

To determine the number average degree of polymerization (*DP*_n_) and the number average molecular weight (*M*_n_) of the synthesized HbPG-PTHF-HbPG block copolymers, and to determine the degree of acetylation of the block copolymers, the block copolymers and the initial PTHF were analyzed by ^1^H NMR spectroscopy. Measurements were performed on a Varian Inova instrument (Varian Inc., Palo Alto, CA, USA) at 500 MHz ^1^H frequency with a 5 mm inverse detection tunable dual-band {^1^H-^19^F}/{^31^P-^15^N} probe using 11 s repetition delay (d1), 3 s acquisition time (at), and 32 transients in deuterated dimethyl sulfoxide (DMSO-*d*_6_) and in CDCl_3_ at room temperature.

#### 3.3.3. Differential Scanning Calorimetry

The thermal behavior of the HbPG-PTHF-HbPG block copolymers and the amine-telechelic PTHF was investigated by differential scanning calorimetry (DSC). The DSC measurements were performed on a Mettler TG50 instrument (Mettler Toledo, Greifensee, Switzerland) in the temperature range of −120–200 °C under nitrogen atmosphere. The first heating cycle was performed to eliminate the thermal history of the samples. The heating rate was 10 °C/min and the nitrogen flow was 50 mL/min. The glass transition temperature (*T*_g_) values were determined as the inflection point of the DSC curves obtained by the second heating cycles. The DSC thermograms of the curcumin and the curcumin-loaded lyophilized polymeric micelle samples were measured using a Mettler Toledo DSC821e instrument (Mettler Toledo, Greifensee, Switzerland) in the temperature range of −80 to 200 °C, with a heating rate of 10 °C/min and a nitrogen flow of 80 mL/min.

#### 3.3.4. Determination of the Critical Micelle Concentration by the Pyrene Probe Method

The critical micelle concentration (*cmc*) of the HbPG-PTHF-HbPG block copolymers was determined by the fluorometric method using pyrene as a fluorescent probe. Pyrene is a hydrophobic fluorescent dye, which exhibits different spectral patterns in micellar and non-micellar solutions due to its sensitivity to the polarity of the medium. A 500 µL volume of a 1.2 mM pyrene solution in acetone was added to a glass flask and dried to form a thin film on the glass surface. Then, 1 L of double-distilled water was added to the flask and stirred overnight to obtain a saturated pyrene solution. A series of polymer solutions with increasing concentrations (5·10^−4^–0.5 g/L) were prepared using the filtered pyrene solution as a medium. The samples were allowed to equilibrate for 4 h. Measurements were performed with a Varian Cary Eclipse fluorescence spectrophotometer (Varian Inc., Palo Alto, CA, USA; right-angle geometry, 1 × 1 cm quartz cell) at 25 °C. The excitation wavelength was 320 nm with a monochromator slit width of 5 nm and an emission slit width of 2 nm. The emission was recorded between 360 and 400 nm. The ratio of the intensities of the first peak (*I*_1_ around 373 nm) and the third peak (*I*_3_ around 383 nm) is a sensitive parameter that characterizes the polarity of the probe’s environment. A low *I*_1_/*I*_3_ value indicates low polarity of the environment. Therefore, *I*_1_/*I*_3_ is expected to decrease at the onset of micelle formation, reflecting preferential solubilization of pyrene into a less polar microenvironment [107,108].

#### 3.3.5. Surface Tension Measurement by the Du Noüy Ring Method

The surface-active behavior of the HbPG-PTHF-HbPG block copolymers was characterized by measuring the surface tension of their aqueous solution by the Du Noüy ring method as a function of the polymer concentration in the range of 10^−4^–5.0 g/L. The *cmc* values were determined by the intersection of the lines fitted to the surface tension versus concentration data.

#### 3.3.6. Dynamic Light Scattering

Dynamic light scattering measurements were performed to determine the *cmc* values of the block copolymers, as well as the particle size and distribution of the obtained micelles. The measurements were performed using a Zetasizer Nano ZS (Malvern Instruments Ltd., Malvern, UK) instrument and a wavelength of 633 nm in disposable semi-micro plastic cuvettes at 25 °C. The derived count rate of the polymer solutions was measured in the range of 0.01–1 g/L. The *cmc* values were defined as the intersection of the lines fitted to the derived count rate versus the polymer concentration data. The size of the HbPG-PTHF-HbPG block copolymer micelles was characterized by measuring the 10 g/L aqueous solutions of the copolymers to determine their intensity mean diameter (*d*) and dispersity index (*Đ*).

#### 3.3.7. Transmission Electron Microscopy

Transmission electron microscopy (TEM) (Zeiss Leo 912 Omega, Oberkochen Germany) was performed on aqueous solution of the ***P2*** sample (*c*_copolymer_ = 5 g/L). Continuous Carbon-Coated Grids were made hydrophilic using a PELCO easiGlow (Ted Pella, Inc., Redding, CA, USA) glow discharger. A 7 µL volume of the sample solution was applied to the carbon grids for 1 min. After blotting, the grids were placed on a drop of 3% uranyl acetate solution and immediately blotted. Then the grid was placed again on a drop of 3% uranyl acetate solution, this time for 30 s. After blotting, the grid was dried for 20 min. Imaging was performed using a Zeiss Leo 912 Omega TEM (Zeiss, Oberkochen, Germany) at 120 kV operating voltage. Images were taken using a 1K camera. A histogram distribution plot of the size of the micelles was made from the determined diameter of 250 individual micelles measured manually using ImageJ software, version 1.54g.

#### 3.3.8. Small-Angle X-Ray Spectroscopy

The shape and hydrodynamic size of the ***P1*** sample were investigated by small-angle X-ray scattering (SAXS) by measuring the aqueous solution of the ***P1*** triblock copolymer (*c*_copolymer_ = 15 g/L). The measurements were performed on the CREDO instrument equipped with a GeniX^3D^ Cu ULD integrated beam delivery system with a 30W microfocus Cu anode X-ray tube and a parabolic FOX^3D^-graded multilayer mirror (Xenocs SA, Sassenage, France), as described elsewhere [109,110]. The samples to be measured were filled into borosilicate glass capillaries of 1.5 mm diameter and 0.01 mm wall thickness, which were placed into an aluminum sample holder block situated in the vacuum chamber of the SAXS instrument. Measurements were performed with a Pilatus-300k CMOS hybrid pixel detector (Dectris Ltd., Baden, Switzerland). In order to cover a wider angular range, two different sample-to-detector distances were used, 531 and 1296 mm. Water was measured as background under the same conditions as the sample. Scattering experiments were performed repetitively: 5-minute exposures were performed on the samples, frequently conducting reference measurements of external and internal background noise (“dark” and “empty beam”, respectively). This was accompanied by two calibrants: a piece of glassy carbon for absolute intensity scaling as well as a mixture of silver behenate and an SBA-15 mesoporous silica for calibrating the horizontal, angular axes. Each distinct scattering pattern was corrected for external and internal background, geometrical distortions (detector flatness), and X-ray absorption on the sample. Intensity was calibrated into instrument-independent units of differential scattering cross-section. The angular dependence of the scattering curves was expressed as the momentum transfer (defined as $q=4\pi sin\left(\theta \right)/\lambda$, where 2θ is the scattering angle and λ is the wavelength of the used Cu Kα radiation, 0.154 nm). An average of the resulting corrected scattering patterns was calculated for each sample measured at each sample-to-detector distance. Scattering curves were obtained by azimuthal averaging. The scattering curves measured at different experimental setups, corresponding to different *q*-ranges, were merged by considering their overlap. Scaling was performed, but the scaling factors were near 1 in all cases due to the absolute intensity calibration mentioned above. To determine the size and geometry of the micelles, a core–shell ellipsoid model was fitted to the measured data using the SasView software, version 5.0.6 (https://www.sasview.org/, accessed on 3 February 2025).

#### 3.3.9. Coagulation Experiments

The effect of temperature on the stability of the HbPG-PTHF-HbPG block copolymers was determined by turbidity measurements. The transmittance of 0.1 g/L polymer PBS solutions at 488 nm was recorded in the 25–80 °C range at a heating rate of 1 °C/min. A Jasco V-650 UV-Vis instrument (JASCO Corporation, Tokyo, Japan) equipped with a Jasco MCG-100 mini circulation bath and a Peltier thermostat heating and cooling system, 1 × 1 cm quartz cuvettes, with distilled water as a reference, was used for the measurements.

### 3.4. Encapsulation and Drug Release Experiments

#### 3.4.1. Solubilization of Curcumin

The curcumin-loaded polymer micelles were prepared by adding 150 μL curcumin solution (20 g/L in acetone) dropwise to 3 mL vigorously stirred aqueous solutions of HbPG-PTHF-HbPG block copolymers, the concentrations of which varied in the range of 0.025–50 g/L. The organic solvent was evaporated by stirring overnight under atmospheric pressure at room temperature. The resulting suspensions were centrifuged (5 min, 3000 rpm), and the supernatants were filtered through 0.45 µm syringe filters to remove the excess curcumin. The amount of curcumin solubilized (encapsulated) by the block copolymers was determined using a Jasco V-650 UV-Vis spectrophotometer (JASCO Corporation, Tokyo, Japan). Then, the required amount of curcumin-loaded block copolymer solutions was added to ethanol and the UV-Vis spectra were recorded at 424 nm in polystyrene semi-micro cuvettes with EtOH reference, using absolute ethanol–water mixture as a reference at 25 °C. The encapsulated drug content was determined based on the calibration curve of curcumin in absolute EtOH (6.78·10^−5^–8.69 × 10^−7^ mol/L, Figure S26). Measurements were performed at least three times. The micelle/water partition coefficient (*P*_M/W_) and the drug loading content (*DLC*) were calculated as follows:

(4) $$P_{M/W}=\frac{c_{CP}-c_{CW}}{c_{CW}}=\frac{c_{CM}}{c_{CW}}$$

(5) $$DLC=\frac{c_{CM}}{c_{CM}+c_{P}}$$

where *c*_CP_ is the measured curcumin concentration in the copolymer solution, *c*_CW_ is the water solubility of curcumin (1.3 mg/L; 3.53 × 10^−6^ mol/L determined by us before the experiments), *c*_CM_ is the concentration of curcumin in the micelles, and the *c*_P_ is the polymer concentration. *DLC* was expressed in weight (*DLC*_g/g_) and molar (*DLC*_mol/mol_) concentrations.

#### 3.4.2. Fluorescence Studies of Curcumin-Loaded Micelles

The fluorescence of curcumin-loaded HbPG-PTHF-HbPG micelles prepared as described above was measured in a phosphate buffer solution (pH = 7) prepared from 180 µL of curcumin-loaded block copolymer aqueous solutions and 2.82 mL buffer (*c*_P1_ = 3 mmol/L, *c*_P2_ = 4 mmol/L, *c*_P3_ = 3 mmol/L, and *c*_curcumin_ = 0.025 mmol/L). Free curcumin was also measured using a sample of 0.025 mmol/L curcumin in a buffer containing 2% DMSO. Each solution was measured using a Varian Cary Eclipse fluorescence spectrophotometer (Varian Inc., Palo Alto, CA, USA; right-angle geometry, 1 × 1 cm quartz cuvette) at 25 °C. The excitation wavelength was 441 nm with a monochromator slit width of 5 nm and an emission slit width of 5 nm, and the emission was recorded between 463 and 750 nm.

#### 3.4.3. Drug–Polymer Interaction Study

To investigate the interaction between the curcumin (***C***) and the block copolymer, a drug-loaded sample (***P1***+***C***) was prepared as described in the solubilization experiments. Briefly, 100 μL curcumin solution (20 g/L in acetone) was added to the HbPG-PTHF-HbPG block copolymer solution (***P1***: 21.9 mg dissolved in 2 mL D_2_O) and was stirred overnight. The suspension was then centrifuged (5 min, 3000 rpm) and the supernatant was filtered through a 0.45 µm syringe filter. The sample was measured on a Varian NMR SYSTEM spectrometer (Varian Inc., Palo Alto, CA, USA) operating at 600 MHz ^1^H frequency, equipped with an inverse three-channel HCX probe and a Z-direction gradient of 65 Gauss/cm. Pulse sequences from Varian’s VnmrJ 4.0 software were used, double-pulsed-field gradient echo (DPFGE) NOESY (NOESY1D) for selective one-dimensional NOE, and NOESY for two-dimensional gradient NOE. One-pulse ^1^H spectra were measured using 16 s repetition delay (d1), 4 s acquisition time (at), and 64 transients. The two-dimensional NOESY spectrum was acquired with 1 s d1 (using grad-90-grad option and zero-quantum filter), with 128 increments in the indirect dimension using 64 transients (4096 points), with 200 ms mixing time. Selective one-dimensional NOESY spectrum was recorded with 1 s d1 (using grad-90-grad option and ZQ filter), 4 s at using 256 transients, with 150 ms mixing time at 25 °C.

#### 3.4.4. Stability of the Curcumin-Loaded Micelles

The size and drug content of curcumin-loaded micelles (*c*_polymer_ = 10 g/L and *c*_curcumin,feed_ = 0.07 g/L) were monitored by DLS (Zetasizer Nano ZS, Malvern Instruments Ltd., Malvern, UK) and UV-Vis spectroscopy (Jasco V-650 spectrophotometer (JASCO Corporation, Tokyo, Japan), 424 nm, 25 °C) for 7 days. Meanwhile, 2 mL of the curcumin-loaded micellar solutions were prepared at the same concentration and lyophilized overnight. The dry samples were stored at room temperature for 3 days and redispersed in 2 mL of water. The size and curcumin content of the micelles were then measured. All measurements were performed in triplicate.

#### 3.4.5. Drug Release Measurements

The curcumin release properties of the HbPG-PTHF-HbPG block copolymers were investigated under two different conditions. In the first case, the drug-loaded micelles were prepared using 25 g/L polymer and 1 g/L curcumin concentrations to obtain maximum encapsulated drug content. First, 1 mL of samples were transferred into dialysis bags (MWCO 1 kDa), sealed, and immersed in 50 mL of PBS solution containing 3% Tween 80, which was stirred and incubated at 37 °C. Then, at predetermined times (0, 2, 4, 6, 8, 24, 48, 72, 96, 168, and 240 h), 50 μL samples were taken from the dialysis bag and diluted with 3 mL EtOH. The curcumin content was followed by UV-Vis spectroscopy using the conditions and equipment as described in Section 3.4.1. In the second case, to study the effect of the composition of the block copolymers, i.e., the HbPG/PTHF ratio, on drug release, similar measurements were performed using the same molar concentration of all block copolymers (0.0017 mol/L) and 0.1 g/L curcumin. Due to the lower drug concentration, 100 μL samples were taken at the initial period (0, 2, 4, 6, 8 h), which is highly important and relevant in biological systems. The samples were added to 2 mL EtOH and measured according to the previously described method. The drug release behavior of the HbPG-PTHF-HbPG block copolymers was compared with a measurement made with a sample prepared by dissolving 0.1 g/L curcumin in DMSO/water (40/60 *V*/*V*%). All measurements were performed at least three times, and the PBS + Tween 80 solution was renewed after each sampling.

### 3.5. Biological Activity of the Curcumin-Loaded HbPG-PTHF-HbPG Micelles

#### 3.5.1. Cytotoxicity Assay

The cytotoxic effect of the micelles was measured on the U-87 glioblastoma cell line (ATCC^®^ HTB-14™) [111,112]. All cellular assays were performed with cells at passages < 15. Before treatment, cells were cultured for 24 h in 10% FBS containing DMEM (15,000 cells/100 µL/well, in a flat-bottom 96-well culture plate). Aqueous stock solutions of the compounds were diluted with media, and a three-fold serial dilution series was prepared (final concentrations: 0.2–500 μM). Cells were treated with the compounds for 3 and 24 h, then cell viability was tested using MTT assay [113,114,115,116]. Briefly, 45 μL MTT solution (2 mg/mL, dissolved in serum-free DMEM) was added to each well. After 2.5 h of incubation, the plates were centrifuged at 2000 rpm for 5 min, and the supernatant was carefully aspirated with a G30 needle. The precipitated purple crystals were dissolved in 100 μL DMSO. After 5 min of agitation, the absorbance was determined at λ = 540 nm and 620 nm using an ELISA plate reader (iEMS Reader, Labsystems, Helsinki, Finnland). Cytotoxicity, expressed in percentage as a function of the compound concentration, was graphically presented and *IC*_50_ values were determined using a GraphPad Prism dose–response fitting.

#### 3.5.2. Assessment of Cellular Uptake by Flow Cytometry

The internalization of curcumin and curcumin-loaded micelles was measured on U-87 glioblastoma cell line. Cells were harvested in the logarithmic growth phase and plated on 24-well tissue culture plates (10^5^ cells/1 mL DMEM/well) 24 h before the experiment. Stock solutions of curcumin and curcumin-loaded micelles were diluted with serum-free DMEM and added to the cells at 12.5 and 25 µM final concentrations. The concentrations were calculated to the curcumin content corresponding to 87.5 and 175 µM polymer concentrations. The cells were incubated with the compounds in a humidified atmosphere containing 5% CO_2_ at 37 °C for 3 h. After centrifugation (1000 rpm, 5 min) and washing the cells twice with the medium, the supernatant was removed and 100 μL of 1 mM trypsin was added to the cells. Trypsinization was stopped after 2 min of incubation by adding 0.8 mL of 10% FCS/HPMI medium (modified HPMI composition [106]; in-house composition: Hepes buffer HPMI (100 mM NaCl, 5.4 mM KCl, 0.4 mM MgCl_2_, 0.04 CaCl_2_, 10 mM Hepes, 20 mM glucose, 24 mM NaHCO_3_, and 5 mM Na_2_HPO_4_) at pH 7.4.); then, the cells were washed and resuspended in 0.3 mL of HPMI. Intracellular fluorescence intensity of the cells was measured using a BD LSR II flow cytometer (BD Biosciences, San Jose, CA, USA) on channel PE LP550 (emission at λ = 550 nm), and the data were analyzed using FACSDiva 5.0 software. All measurements were performed in triplicate, and the mean fluorescence intensity (MFI) and the percentage of FITC-positive cells were graphically presented. The percentage of live cells was compared to untreated control cells to assess relative viability. Data are mean ± SEM. To analyze statistical significance (p), the one-way ANOVA, followed by Tukey’s post hoc test was performed using GraphPad Prism software, version 8.0.2.

#### 3.5.3. Atomic Force Microscopy

To prepare samples for AFM imaging, round glass coverslips (12 mm) were autoclaved, and the cells were seeded on them in a 24-well tissue culture plate (cell number was adjusted to 6 × 10^5^ cells/mL). The cells were allowed to attach overnight in 5% CO_2_ at 37 °C, and then treated with the compounds at 25 µM curcumin concentration. After 3 h of incubation, the cells were washed three times with PBS. Before imaging, cells were fixed with 4% glutaraldehyde solution (freshly prepared in PBS) overnight at 4 °C and washed thrice with PBS and distilled water, respectively. High-resolution imaging of fixed and air-dried cells was performed using a Nanosurf Flex-Axiom AFM system (Nanosurf, Liestal, Switzerland) operating in contact mode with a NanoWorld CONTR silicon cantilever (NanoWorld, Neuchâtel, Switzerland) with a force constant of 0.2 N/m. The membrane roughness of each sample was imaged and determined in 2 × 2 µm^2^ areas on 10 randomly selected cells. Average roughness parameters were collected for at least 3000 line profiles. The surface roughness, *R*_a_ is the average deviation of all points of the roughness profile from an average line over the evaluation length:

(6) $$R_{a}=\frac{1}{N}\sum_{j=1}^{N}\left|r_{j}\right|$$

## 4. Conclusions

Amphiphilic ABA triblock copolymers composed of hyperbranched polyglycerol (HbPG) and poly(tetrahydrofuran) (PTHF) were synthesized by ring-opening, multibranching polymerization of glycidol using amine-telechelic PTHF as macroinitiator. We found that the macroinitiator was completely incorporated into the block copolymers, and the formation of the designed hyperbranched–linear–hyperbranched structure was confirmed by GPC, ^1^H NMR, and DSC results. In addition, DSC measurements revealed that the crystallization of the PTHF segment in the block copolymers is hindered by the hydrophilic HbPG blocks.

Based on the amphiphilic structure of the prepared macromolecules, surface-active characteristics were expected. The results of the fluorometric pyrene probe, surface tension, and DLS measurements confirmed the self-assembly behavior of the block copolymers with *cmc* values in the range of 0.1–0.5 g/L. Formation of micellar-type aggregates about 15 nm in size was observed above the *cmc* by independent measurements (DLS, TEM, SAXS). The results of turbidimetric measurements show that the nanomicelles possess high colloidal stability against temperature changes, providing that these materials can be used in biological systems without coagulation.

Amphiphilic block copolymers can potentially be used as nanosized drug delivery systems due to their beneficial encapsulation properties of hydrophobic drugs. Therefore, the bio-applicability of the prepared ABA triblock copolymers was investigated using a highly water-insoluble hydrophobic natural drug, curcumin. Highly efficient solubilization (encapsulation) of curcumin, up to 1500-fold increase, was achieved, which can be influenced by the composition of the block copolymers. The in vitro drug release experiments showed a sustained profile, highly dependent on the drug content and slightly dependent on the size of the HbPG blocks. In addition, high stability of the curcumin-loaded micelles under storage and lyophilization was found, i.e., no changes in the drug content or the size were observed. The bioactivity assays with U-87 human glioblastoma cell line showed that the internalization rates of the curcumin-loaded block copolymers were significantly higher than the internalization rate of free curcumin. In addition, none of the curcumin-loaded micelles showed relevant cytotoxicity in the concentration range and incubation time applied. The average surface roughness of the treated cells is reduced by using the block copolymer with the highest HbPG content. The results of the cellular assays indicate that the ***P1*** polymer has the greatest ability to deliver cargos (drug candidates, imaging moieties, etc.), but this compound has moderate cytotoxicity. The ***P3*** polymer has no cytotoxic effect up to 500 µM treatment concentration but is associated with lower efficacy as a carrier. Therefore, the most promising of the HbPG-PTHF-HbPG block copolymer series produced for drug delivery systems is ***P2***, which contains the hydrophobic and hydrophilic segments in a 1:2 ratio. As a consequence, due to the structure of the HbPG-PTHF-HbPG triblock copolymers, the self-assembly and drug delivery properties can be influenced by the composition, i.e., the *cmc* and drug release can be controlled by the relative amount of HbPG—and the drug encapsulation is primarily determined by the molarity of the hydrophobic PTHF segment in these block copolymers.

Based on our results, it can be concluded that the novel series of the HbPG-PTHF-HbPG ABA triblock copolymers are suitable drug delivery systems for the solubilization (encapsulation) of various biomaterials, such as drugs, dyes, and theranostic agents. They can also be utilized as surfactants for the preparation of nanoparticles and nanocomposites. In addition, the hydroxyl groups of the HbPG segments of these block copolymers can be further functionalized, opening new routes for synthesizing targeted drug delivery systems.

## Figures and Tables

**Table 1:** Characterization results of the HbPG-PTHF-HbPG amphiphilic block copolymers: theoretical (*M*_theor_) and measured (*M*_n_) number average molecular weights determined by ^1^H NMR and GPC, dispersity index (*Đ*), and the glass transition temperature values of the PTHF (*T*_g_^1^) and HbPG (*T*_g_^2^) segments.

Polymer	*M*_theor_ (g/mol)	*M*_n_ (g/mol)			*Đ*	*T_g_* ^1^	*T_g_* ^2^
		^1^H NMR ^a^	^1^H NMR ^b^	GPC		(°C)	
***P1***	2200	2190	2320	4000	1.47	−77	−43
***P2***	3300	3410	3500	5500	1.51	−74	−32
***P3***	5500	5890	6020	7000	1.64	−79	−30

^a^ determined from the ^1^H NMR spectra of the unmodified block copolymers. ^b^ determined from the ^1^H NMR spectra of the acetylated block copolymers.

**Table 2:** The critical micelle concentrations (*cmc*) of the HbPG-PTHF-HbPG block copolymers determined by the pyrene probe method, the surface tension measurement, the Du Noüy ring method, and by DLS, as well as the average micellar hydrodynamic diameters (*d*) and dispersity values (*Đ*) obtained by DLS.

Polymer	PTHF Content (m/m%)	*cmc*						Micellar Size	
		Pyrene Probe		Surface Tension		DLS			
		(g/L)	(µmol/L)	(g/L)	(µmol/L)	(g/L)	(µmol/L)	*d* (nm)	*Ð*
***P1***	55	0.11	50.2	0.20	91.3	0.18	82.2	13.2	0.056
***P2***	35	0.13	38.1	0.34	99.7	0.21	61.4	13.1	0.070
***P3***	21	0.18	30.6	0.56	95.1	0.24	40.7	14.8	0.133

**Table 3:** Curcumin solubilization (encapsulation) characteristics of the HbPG-PTHF-HbPG block copolymers: solubilized curcumin concentration (mass and molar), the micelle/water partition coefficients (*P*_M/W_), and the drug loading contents (*DLCs)* by using 50 g/L polymer solution.

Polymer	*c*_curcumin_		*P*_M/W_	*DLC* (%)	
	(g/L)	(mmol/L)		(g/g)	(mol/mol)
***P1***	1.72 ± 0.15	4.68 ± 0.42	1570 ± 139	3.3 ± 0.3	17.0 ± 1.3
***P2***	0.92 ± 0.30	2.48 ± 0.83	833 ± 277	1.8 ± 0.6	14.3 ± 4.0
***P3***	0.60 ± 0.05	1.64 ± 0.13	550 ± 44	1.2 ± 0.1	16.2 ± 1.1

**Table 4:** The average surface roughness of cells treated with curcumin and curcumin-loaded nanomicelles.

Sample	*R*_α_ (nm)
untreated cell	14.2 ± 4.8
curcumin-treated cell	11.4 ± 3.5
***P1*** + ***C***-treated cell	10.5 ± 3.7
***P2*** + ***C***-treated cell	13.5 ± 4.8
***P3*** + ***C***-treated cell	5.7 ± 3.2

## Data Availability

The original contributions presented in this study are included in the article/Supplementary Materials. Further inquiries can be directed to the corresponding authors.

## Supplementary Materials

The following supporting information can be downloaded at: https://www.mdpi.com/article/10.3390/ijms26125866/s1.
