# Supplementary material for: Self-Assembling Amphiphilic ABA Triblock Copolymers of Hyperbranched Polyglycerol with Poly(tetrahydrofuran) and Their Nanomicelles as Highly Efficient Solubilization and Delivery Systems of Curcumin
Source: Int J Mol Sci. 2025 Jun 19;26(12):5866. doi: 10.3390/ijms26125866 (PMC12192588; doi:10.3390/ijms26125866)
Supplement: Supplementary file 1 [file ijms-26-05866-s001.zip › ijms-3664465-supplementary.pdf]

Supplementary Materials

Self-Assembling Amphiphilic ABA Triblock Copolymers of Hyperbranched Polyglycerol with Poly(tetrahydrofuran) and Their Nanomicelles as Highly Efficient Solubilization and Delivery Systems of Curcumin

Dóra Fecske [1,2], György Kasza [1,*], Gergő Gyulai [3], Kata Horváti [4], Márk Szabó [5], András Wacha [6], Zoltán Varga [6], Györgyi Szarka [1], Yi Thomann [7,9], Ralf Thomann [7,9], Rolf Mülhaupt [7,8,9], Éva Kiss [3], Attila Domján [5], Szilvia Bősze [10,11], and Béla Iván [1,*]

1 Polymer Chemistry and Physics Research Group, Institute of Materials and Environmental Chemistry, HUN-REN Research Centre for Natural Sciences, Magyar tudósok körútja 2, H-1117 Budapest, Hungary

2 Hevesy György Doctoral School of Chemistry, ELTE Eötvös Loránd University, Pázmány Péter sétány 1/A, H-1117 Budapest, Hungary

3 Laboratory of Interfaces and Nanostructures, Institute of Chemistry, Eötvös Loránd University, P.O. Box 32, H-1518 Budapest, Hungary

4 MTA–HUN-REN "Momentum" Peptide-Based Vaccines Research Group, Institute of Materials and Environmental Chemistry, HUN-REN Research Centre for Natural Sciences, Magyar tudósok körútja 2., H-1117 Budapest, Hungary

5 NMR Research Laboratory, Centre for Structural Science, HUN-REN Research Centre for Natural Sciences, Magyar tudósok körútja 2., H-1117 Budapest, Hungary

6 Biological Nanochemistry Research Group, Institute of Materials and Environmental Chemistry, HUN-REN Research Centre for Natural Sciences, Magyar tudósok körútja 2., H-1117 Budapest, Hungary

7 Freiburg Materials Research Center, University of Freiburg, Stefan-Meier-Str. 21, D-79104 Freiburg, Germany

8 Freiburg Center for Interactive Materials and Bioinspired Technologies (FIT), University of Freiburg, Georges-Köhler-Allee 105, D-79110 Freiburg, Germany

9 Institute for Macromolecular Chemistry, University of Freiburg, Stefan-Meier-Str. 31, D-79104 Freiburg, Germany

10 HUN-REN–ELTE Research Group of Peptide Chemistry, Hungarian Research Network, Pázmány Péter sétány 1/A, H-1117 Budapest, Hungary

11 Department of Genetics, Cell- and Immunobiology, Semmelweis University, Faculty of Medicine, Nagyvárad tér 4, H-1089 Budapest, Hungary

* Correspondence: kasza.gyorgy@ttk.hu (G.K.); ivan.bela@ttk.hu (B.I.)

Table S1. The hydrodynamic size (d) and dispersity ($Đ$) and the curcumin content of the curcumin-loaded micelles during storage for 7 days and after lyophilization and redispersion.

	Polymer	d (nm)	$Đ$	$c_{curc.}$ (g/L)
P1	unloaded	13.2	0.056	-
P1+C	curcumin-loaded	13.6	0.040	0.047 ± 0.002
	stored 1 day	13.8	0.043	0.050 ± 0.003
	stored 7 days	13.5	0.039	0.040 ± 0.002
	lyophilized and redispersed	15.0	0.327	0.042 ± 0.002
P2	unloaded	13.1	0.070	-
P2+C	curcumin-loaded	15.4	0.089	0.054 ± 0.002
	stored 1 day	16.3	0.103	0.048 ± 0.001
	stored 7 days	15.4	0.082	0.052 ± 0.004
	lyophilized and redispersed	15.5	0.507	0.044 ± 0.002
P3	unloaded	14.8	0.133	-
P3+C	curcumin-loaded	17.9	0.138	0.045 ± 0.002
	stored 1 day	18.2	0.132	0.047 ± 0.003
	stored 7 days	17.7	0.108	0.050 ± 0.004
	lyophilized and redispersed	15.9	0.293	0.042 ± 0.003

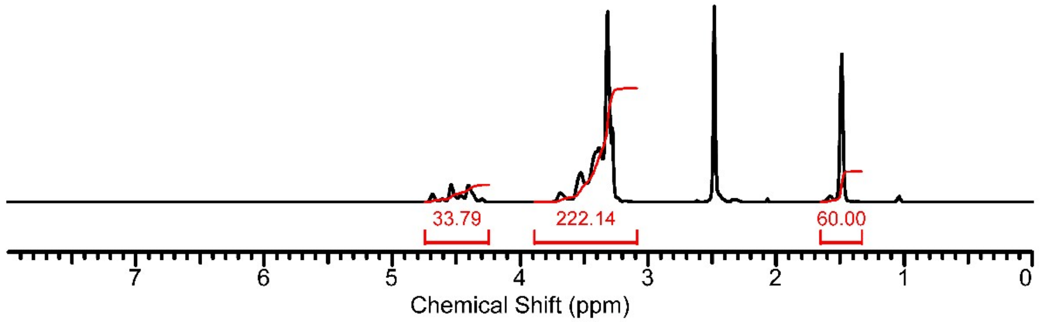

Figure S1. The ^{1}H NMR spectrum of the HbPG-PTHF-HbPG block copolymer sample ***P2*** (DMSO-d_6).

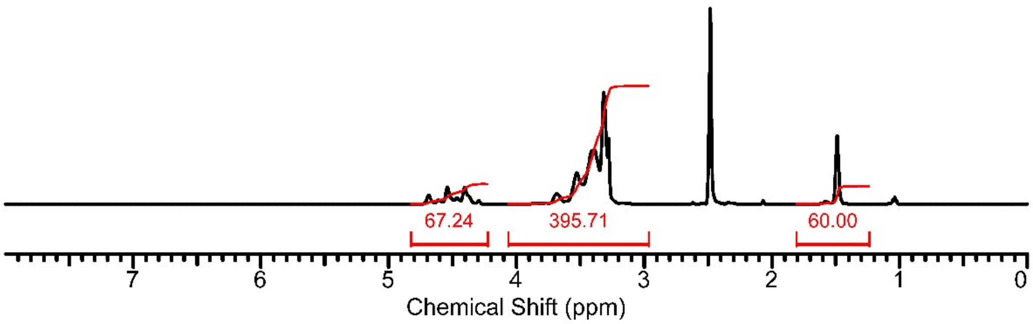

Figure S2. The ^{1}H NMR spectrum of the HbPG-PTHF-HbPG block copolymer sample ***P3*** (DMSO-*d*6).

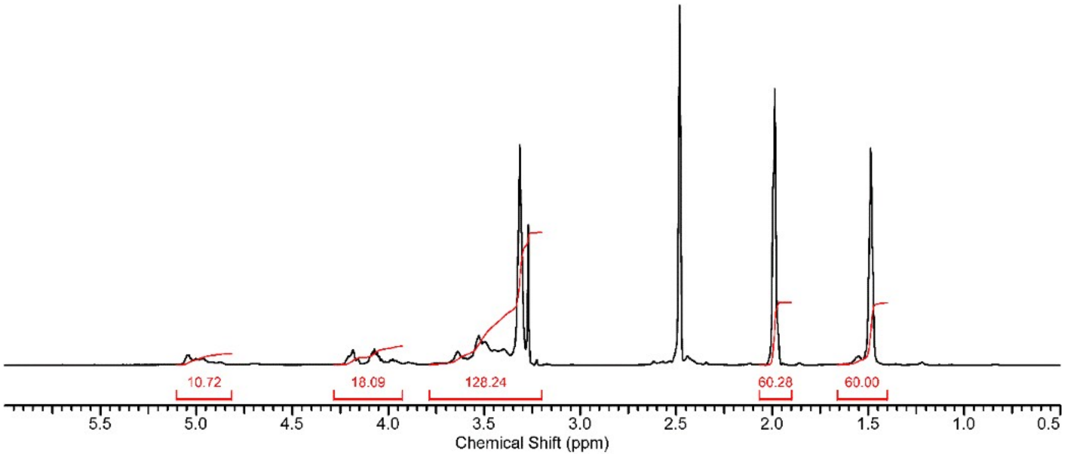

Figure S3. The ^{1}H NMR spectrum of the acetylated HbPG-PTHF-HbPG block copolymer sample ***P1*** (DMSO-*d*6).

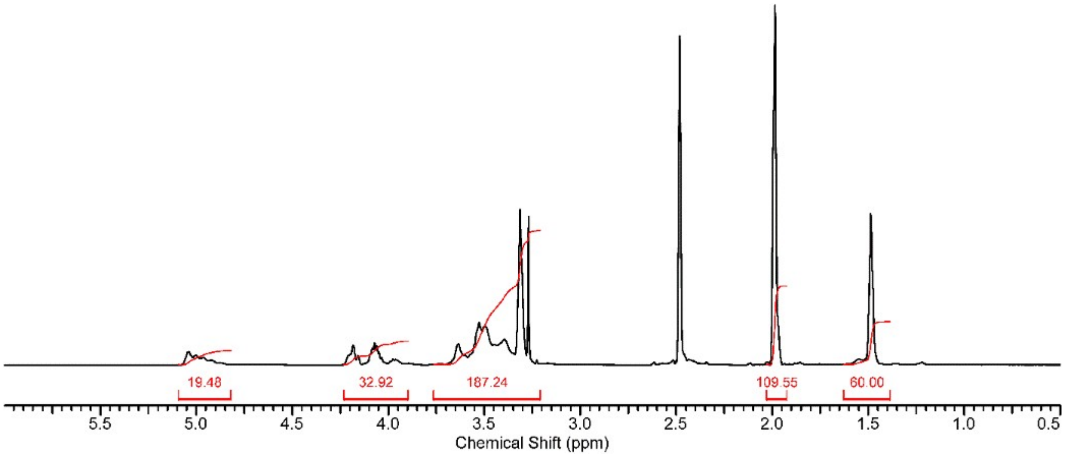

Figure S4. The ^{1}H NMR spectrum of the acetylated HbPG-PTHF-HbPG block copolymer sample ***P2*** (DMSO-*d*6).

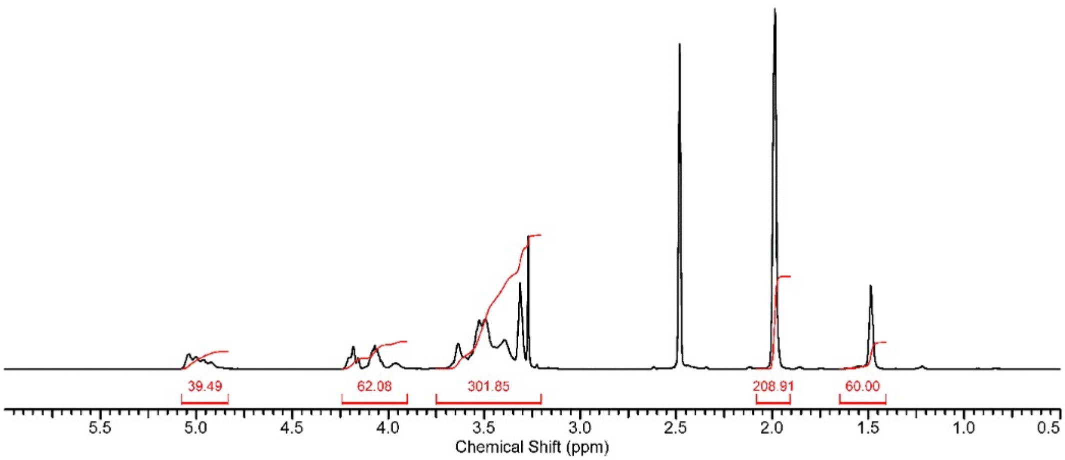

Figure S5. The ^{1}H NMR spectrum of the acetylated HbPG-PTHF-HbPG block copolymer sample ***P3*** (DMSO-d_6).

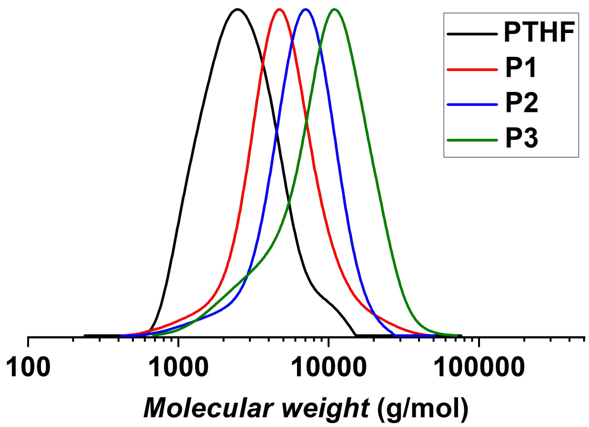

Figure S6. The molecular weight distribution curves of the HbPG-PTHF-HbPG amphiphilic block copolymers and the PHTF macroinitiator.

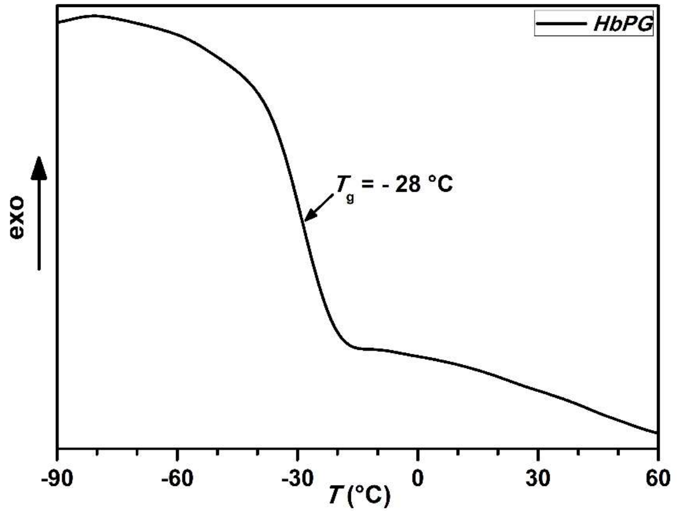

Figure S7. The DSC curve of the HbPG (second heating).

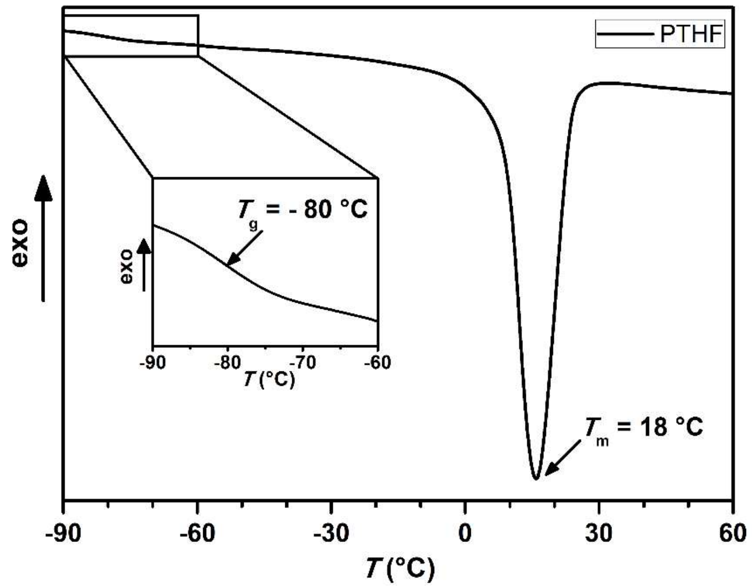

Figure S8. The DSC curve of the initial amine-telechelic PTHF (second heating).

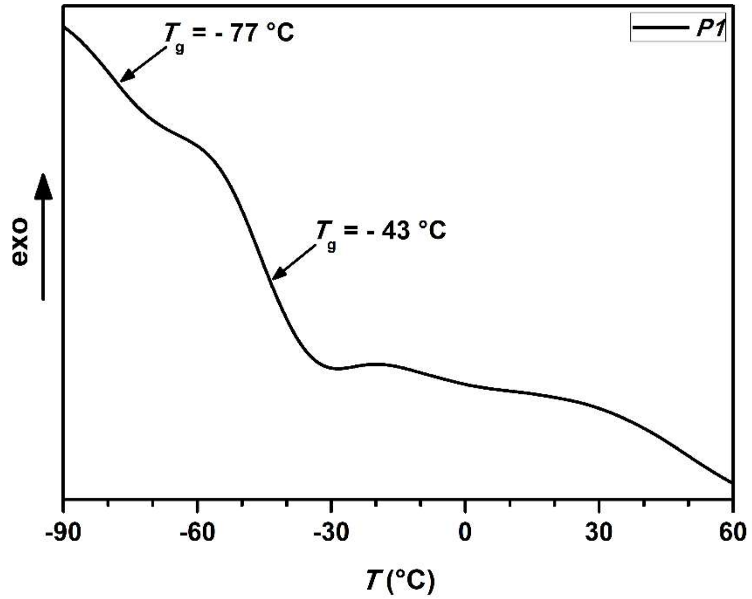

Figure S9. The DSC curve of the HbPG-PTHF-HbPG amphiphilic block copolymer sample ***P1*** (second heating).

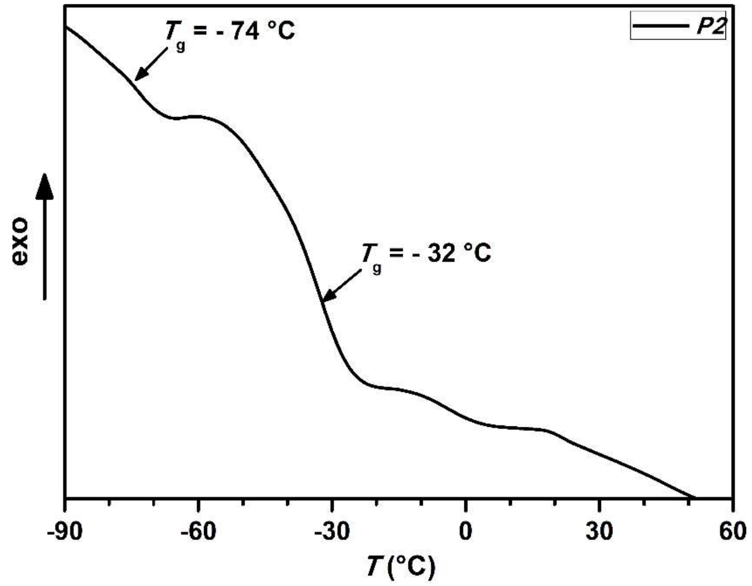

Figure S10. The DSC second heating curve of the HbPG-PTHF-HbPG amphiphilic block copolymer sample ***P2*** (second heating).

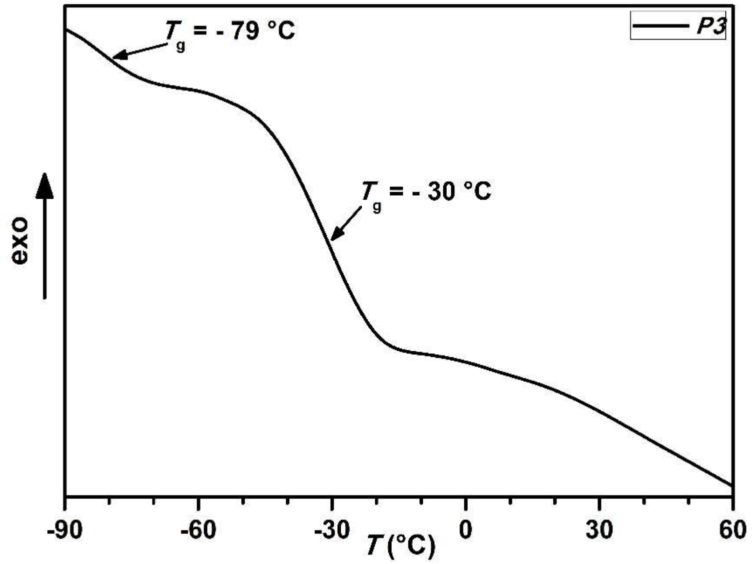

Figure S11. The DSC second heating curve of the HbPG-PTHF-HbPG amphiphilic block copolymer sample ***P3*** (second heating).

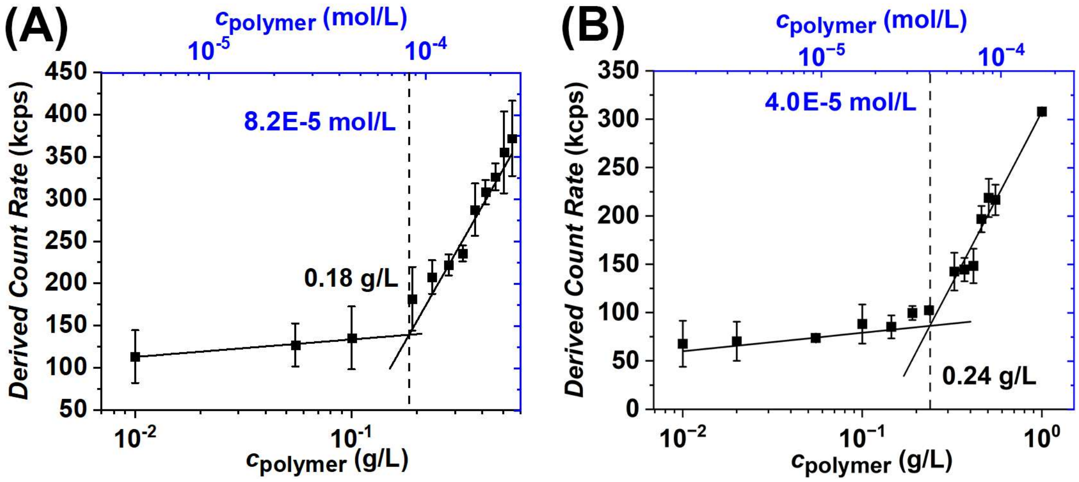

Figure S12. Derived count rate of the aqueous solutions of the HbPG-PTHF-HbPG amphiphilic block copolymers determined by DLS (**A**: ***P1;*** **B**: ***P3***) as a function of the polymer concentration (the *cmc* values are marked with dashed line).

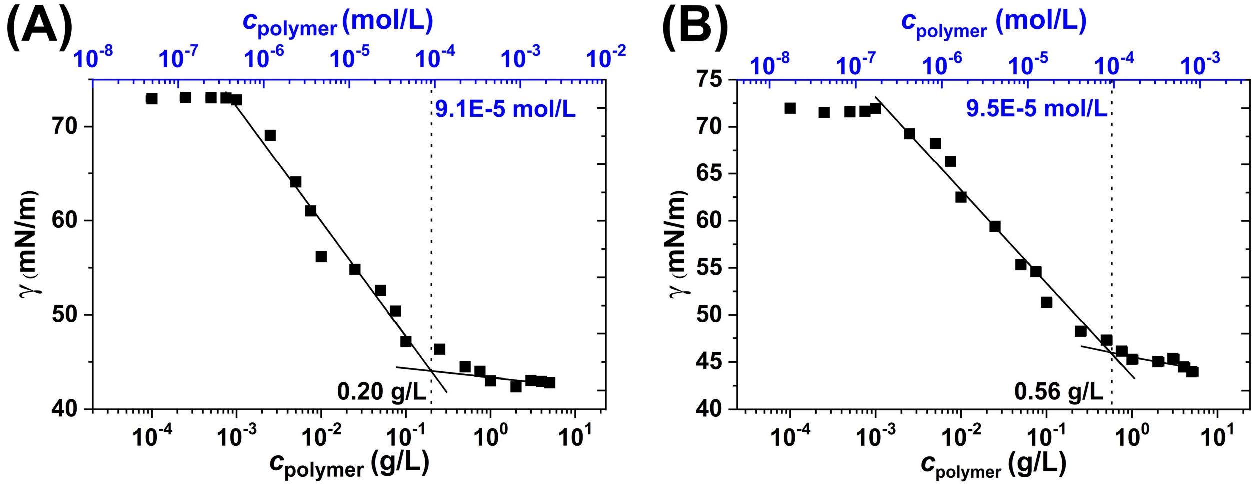

Figure S13. The surface tension of the aqueous solutions of the HbPG-PTHF-HbPG amphiphilic block copolymers (**A**: ***P1;*** **B**: ***P3***) as a function of the polymer concentration (the *cmc* values are marked with dashed line).

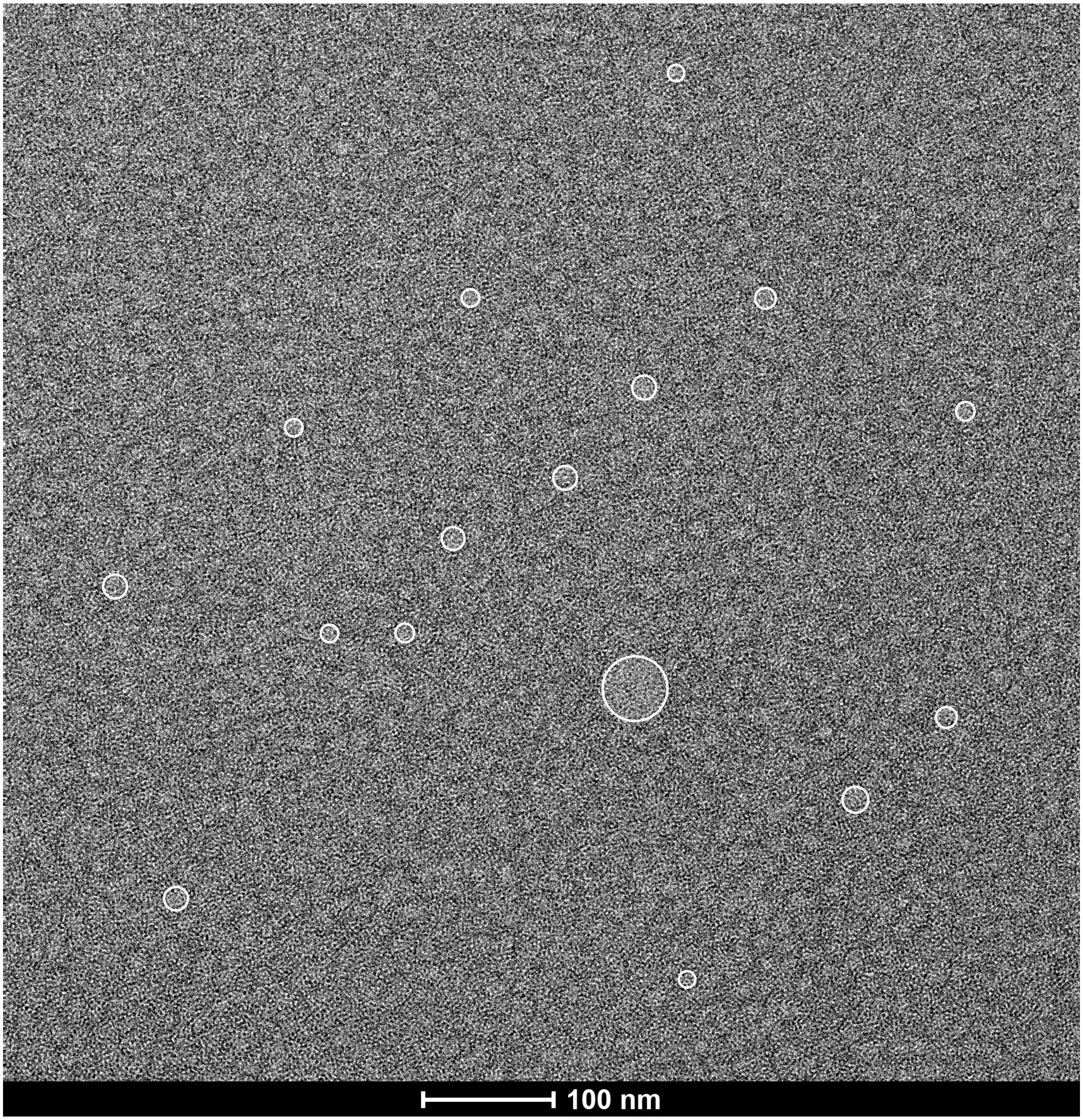

Figure S14. The enlarged TEM image of the ***P2*** block copolymer-based micelles.

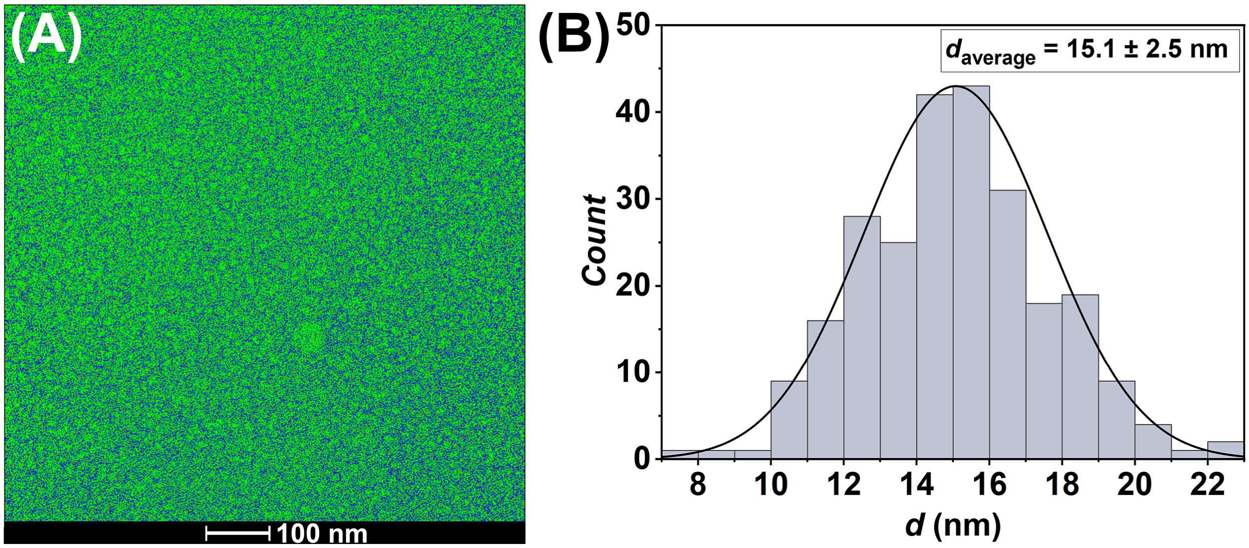

Figure S15. TEM image (**A**) (ImageJ software was used to determine the size of the micelles, for better visualization the threshold values were adjusted to below: 24%, above: 53%) and histogram distribution plot of 250 micelles of ***P2*** block copolymer sample (**B**).

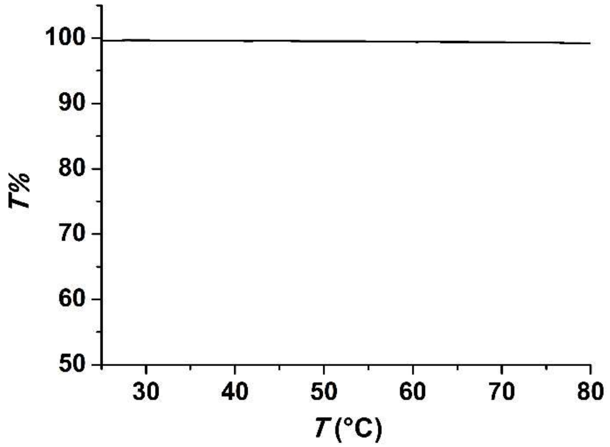

Figure S16. The transmittance versus temperature curve of the ***P1*** HbPG-PTHF-HbPG sample (488 nm, 25-80 °C, 1 °C/min; polymer concentration: 0.1 g/L in water).

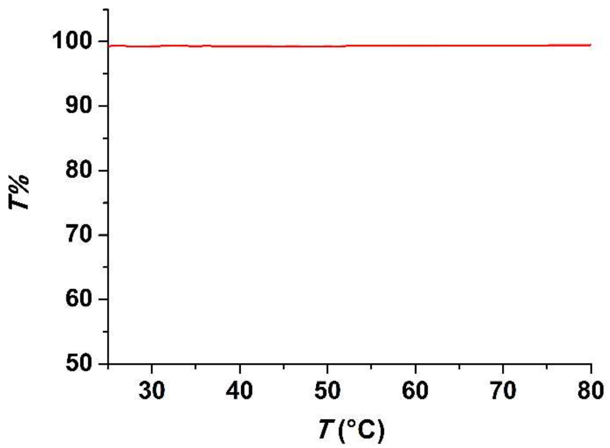

Figure S17. The transmittance versus temperature curve of the ***P2*** HbPG-PTHF-HbPG sample (488 nm, 25-80 °C, 1 °C/min; polymer concentration: 0.1 g/L in water).

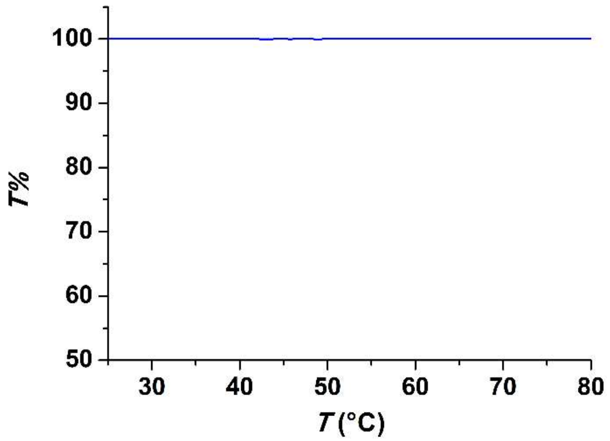

Figure S18. The transmittance versus temperature curve of the ***P3*** HbPG-PTHF-HbPG sample (488 nm, 25-80 °C, 1 °C/min; polymer concentration: 0.1 g/L in water).

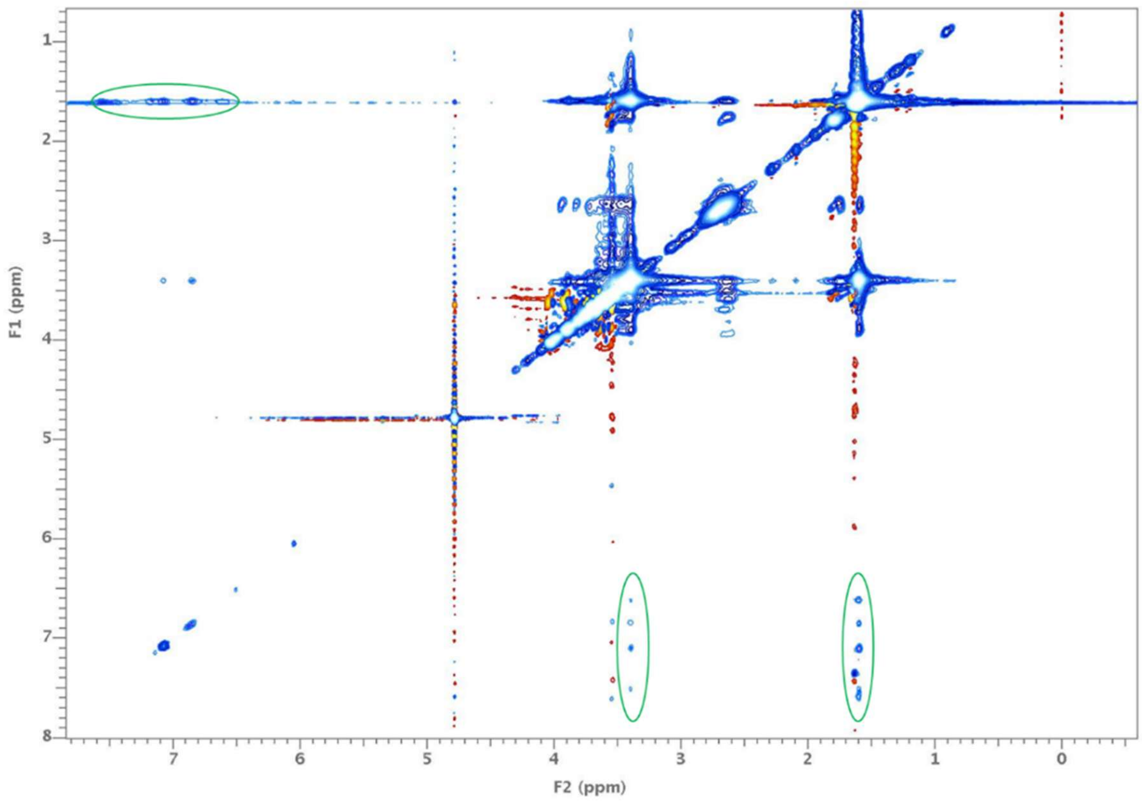

Figure S19. The ^{1}H NOESY spectrum of the curcumin-loaded HbPG-PTHF-HbPG micelles (***P1***) in D_2O (relevant cross-peaks are marked by green ellipses).

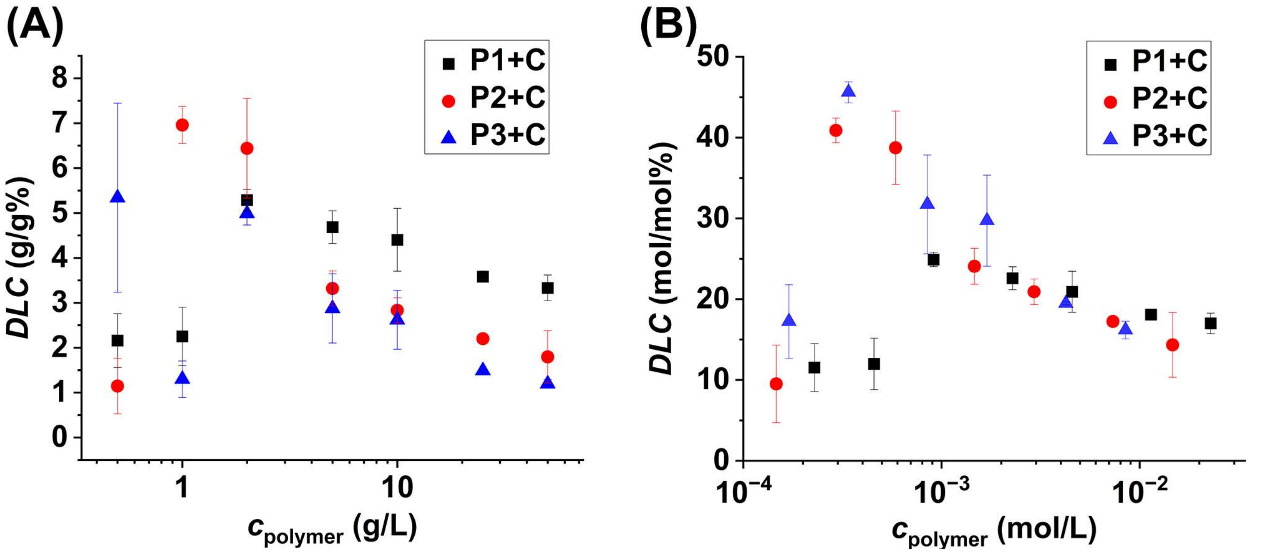

Figure S20. The drug loading content (*DLC*) by weight (**A**) and moles (**B**) as a function of polymer concentration.

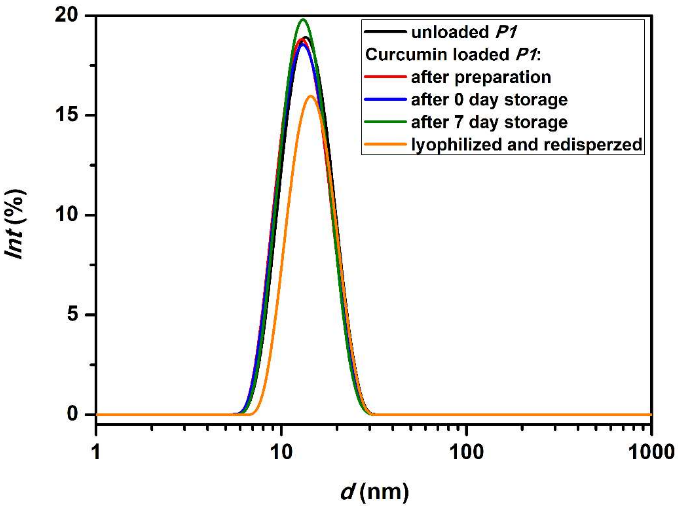

Figure S21. The DLS curves of the unloaded and curcumin-loaded ***P1*** HbPG-PTHF-HbPG amphiphilic block copolymer sample.

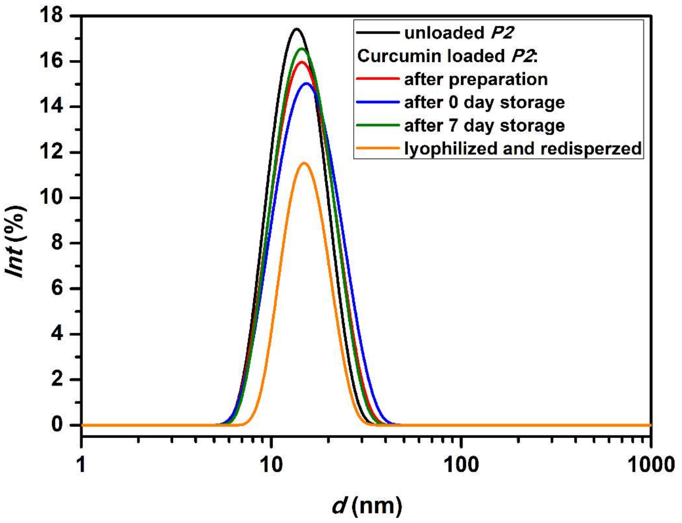

Figure S22. The DLS curves of the unloaded and curcumin-loaded ***P2*** HbPG-PTHF-HbPG amphiphilic block copolymer sample.

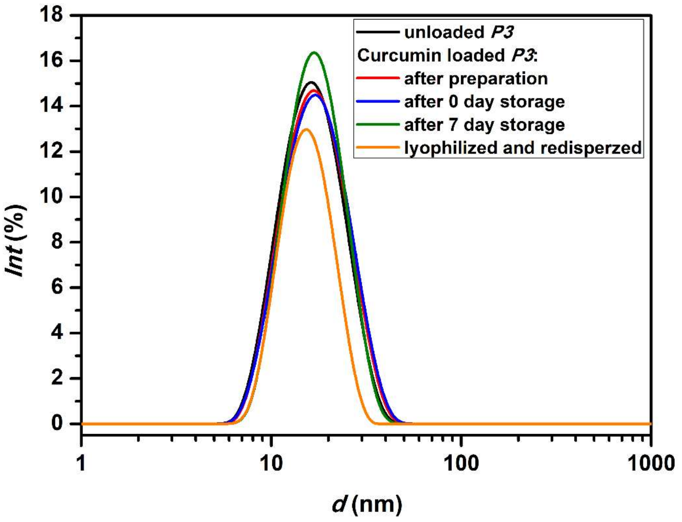

Figure S23. The DLS curves of the unloaded and curcumin-loaded ***P3*** HbPG-PTHF-HbPG amphiphilic block copolymer sample.

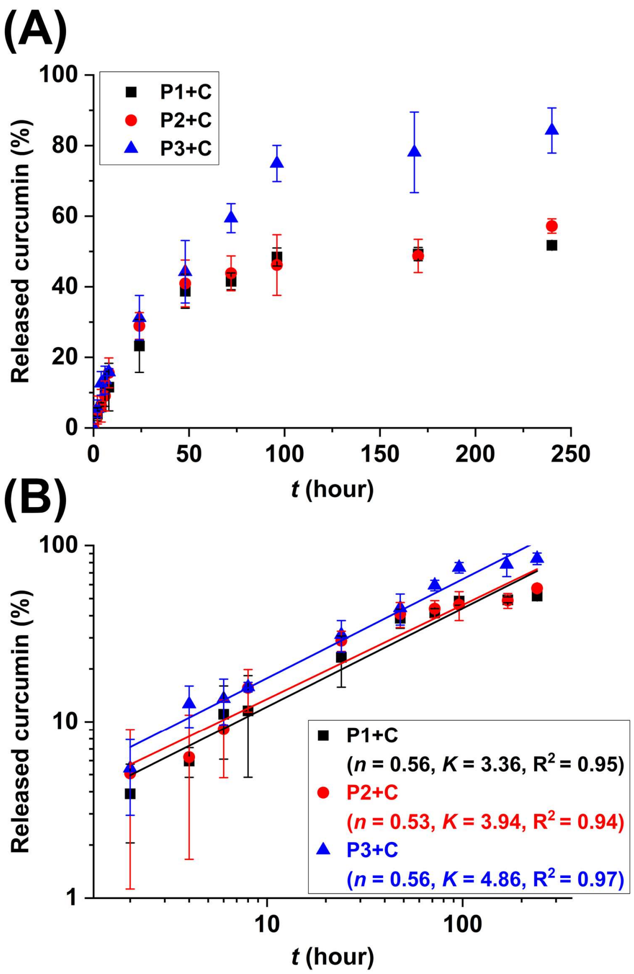

Figure S24. The released amount of curcumin (*R*) from HbPG-PTHF-HbPG copolymers as a function of time (**A**), and the log–log plot of the released amount of the drug and time (**B**) according to the Korsmeyer-Peppas equation ($R=K\cdot t^n$) [99].

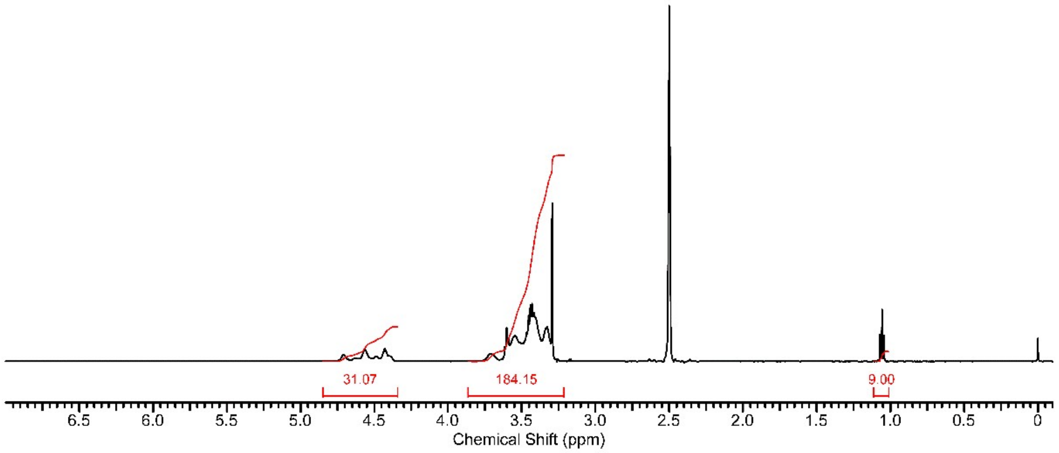

Figure S25. The ^{1}H NMR spectrum of the HbPG sample prepared by neopentyl alcohol initiator (DMSO-*d*6).

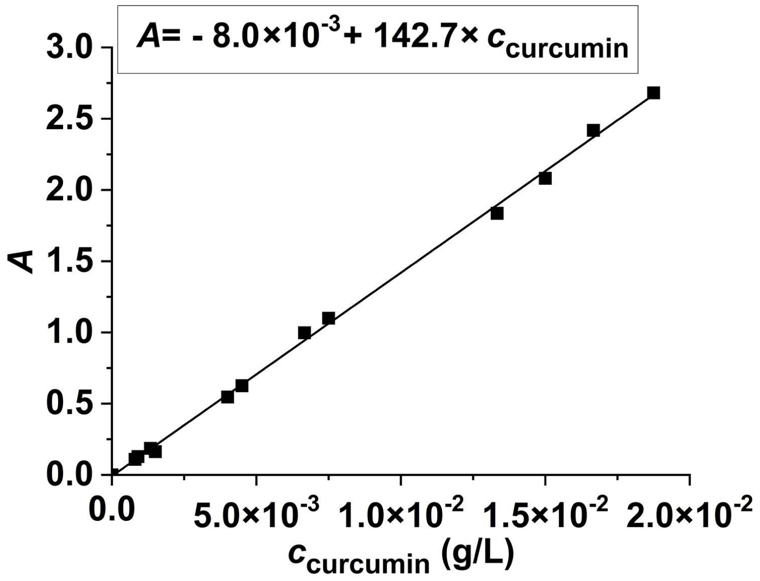

Figure S26. Absorbance values of curcumin solutions in ethanol as a function of curcumin concentration and the calibration curve equation.
